# Difficult-to-treat resistance, not carbapenem non-susceptibility, is associated with 30-day mortality in respiratory gram-negative bacilli: a 12-year surveillance cohort

**DOI:** 10.3389/fcimb.2026.1871310

**Published:** 2026-07-20

**Authors:** Yahya Shabi, Nawal Alwadei, Mona Alshahrani, Ali M. Somily, Khalifa Binkhamis, Elham Asiri, Soliuman Mohamed, Abdulah O. S. Bawazeer, Saud Mushabbab Al Oudhah, Rawan Abdullah Alqahtani, Rawan Alhamlan, Eman Salem, Basma Al Ghamdi, Ghadah Habtar, Amal Alqahtani, Fatimah Asiri, Mazen Jali, Hussein Almahdi, Hana Alahmari

**Affiliations:** 1 Department of Microbiology and Clinical Parasitology, College of Medicine, King Khalid University, Abha, Saudi Arabia; 2 Department of Pulmonology, Aseer Central Hospital, Abha, Saudi Arabia; 3 Department of Medicine, College of Medicine, King Khalid University, Abha, Saudi Arabia; 4 Department of Pathology, College of Medicine, King Saud University and King Saud University Medical City, Riyadh, Saudi Arabia; 5 Department of Pathology, College of Medicine, King Saud University, Riyadh, Saudi Arabia; 6 Internal Medicine Residency Program, Aseer Central Hospital, Abha, Saudi Arabia; 7 Department of Haematology and Immunohaematology, Faculty of Medical Laboratory Sciences, University of Khartoum, Khartoum, Sudan; 8 Department of Microbiology, Aseer Central Hospital, Abha, Saudi Arabia

**Keywords:** antimicrobial resistance, difficult-to-treat resistance (DTR), gram-negative bacilli, mortality, respiratory infections

## Abstract

**Objectives:**

Antimicrobial resistance in respiratory gram-negative bacilli (GNB) is rising in Saudi Arabia, yet the clinical impact of difficult-to-treat resistance (DTR) remains poorly defined. We characterized temporal trends in organism distribution, non-susceptibility phenotypes, and their association with 30-day in-hospital mortality.

**Methods:**

Retrospective cohort of hospitalized patients aged ≥12 years with culture-positive respiratory GNB at a referral hospital in southern Saudi Arabia (January 2013–August 2024). Non-susceptibility phenotypes were fluoroquinolone (FQ-NS), extended-spectrum cephalosporin (ESC-NS), carbapenem non-susceptibility, and DTR. Trends were assessed with segmented binomial regression, mortality with modified Poisson regression.

**Results:**

Among 6,999 isolate-episodes from 3,808 patients, Enterobacterales (57.2%) increased while non-fermenting GNB declined (AAPC, −2.5%). Carbapenem non-susceptibility rose among Enterobacterales (AAPC, + 5.4%), driven by *Klebsiella pneumoniae*. DTR was 37.6%, rising in Enterobacterales (AAPC, + 12.1%) but declining in *Acinetobacter baumannii*. Thirty-day mortality was 27.9%. DTR was associated with mortality in Enterobacterales (aRR, 1.34; 95% CI, 1.16–1.55) and non-fermenting GNB (aRR, 1.28; 95% CI, 1.12–1.46); carbapenem non-susceptibility without DTR was not. Mortality rose as active first-line agents decreased (*P*-trend<.001).

**Conclusions:**

Respiratory GNB epidemiology is shifting toward Enterobacterales with rising resistance. DTR, not carbapenem non-susceptibility alone, was consistently associated with 30-day mortality and provides a meaningful framework for risk stratification and resistance-centered surveillance.

## Introduction

Antimicrobial resistance (AMR) is a major global health threat that undermines the effective treatment of infections and increases morbidity, mortality, and healthcare burden worldwide (World Health Organization, 2023). Gram-negative bacilli (GNB), including *Escherichia coli, Klebsiella pneumoniae, Pseudomonas aeruginosa*, and *Acinetobacter baumannii*, are of particular concern due to the prominent role of β-lactamase production in mediating resistance (Zowawi et al., 2013).

Respiratory infections, particularly hospital-acquired and ventilator-associated pneumonia, are commonly caused by gram-negative organisms in intensive care settings (Abdalla et al., 2023; Centers for Disease Control and Prevention, 2026), shaped by healthcare exposures and antimicrobial use that drive resistance (World Health Organization, 2015). Respiratory cultures remain central to surveillance and stewardship (Abdalla et al., 2023).

The rising prevalence of fluoroquinolone, extended-spectrum cephalosporin, and carbapenem non-susceptibility has complicated empiric and targeted therapy (Elixhauser et al., 1998; Zowawi et al., 2013). Difficult-to-treat resistance (DTR), defined as non-susceptibility to all first-line agents, identifies infections with severely limited therapeutic options and has been linked to increased mortality (Kadri et al., 2018).

In Saudi Arabia, resistance continues to rise among gram-negative organisms of high clinical importance (Zowawi, 2016), with molecular studies showing predominance of OXA-48-like carbapenemases among *Klebsiella pneumoniae* (Alkhelaiwi et al., 2026).

Longitudinal surveillance from southern Saudi Arabia reports rising carbapenem resistance among *Klebsiella* species (12.4% to 69.3%) and DTR prevalence of 50.7%, with OXA-48-like and NDM as dominant genotypes (Shabi et al., 2026), reinforcing the need for integrated phenotypic and genotypic surveillance.

We therefore characterized temporal trends in organism distribution and non-susceptibility among respiratory GNB and evaluated the association between non-susceptibility phenotypes particularly DTR and 30-day in-hospital mortality.

## Materials and methods

### Study design and setting

This retrospective cohort study was conducted at Aseer Central Hospital, a 500-bed tertiary referral center in southern Saudi Arabia. Hospitalized patients aged ≥12 years with respiratory cultures yielding gram-negative bacilli were included. The cohort comprises culture-confirmed respiratory gram-negative bacilli episodes in hospitalized patients; the unit of analysis is the isolate-episode rather than a clinically adjudicated infection. As a culture-based surveillance study, formal clinical or radiographic diagnostic criteria for respiratory infection (e.g., CDC/NHSN HAP/VAP definitions) were not applied. The protocol was approved by the Institutional Review Board of the Aseer Health Cluster (IRB #F7-2-2025) with a waiver of informed consent for retrospective use of de-identified data. Reporting followed the STROBE guidelines (von Elm et al., 2008).

### Data source and study population

#### Data extraction and study period

Culture-positive respiratory isolates and AST results were extracted from the hospital microbiology database (institutional AMR surveillance program), together with demographics, specimen and culture data, and admission-related clinical information. The study period spanned January 1, 2013 – August 27, 2024 (12 annual time points); the end date reflects an electronic health record migration that precluded later retrieval.

#### Inclusion and exclusion criteria

Eligible isolates were GNB (Enterobacterales or non-fermenting) recovered from respiratory specimens (sputum, endotracheal aspirate, tracheal secretions, or bronchoalveolar lavage). Analyses were restricted to hospitalized patients; outpatient specimens and patients <12 years were excluded (eMethods). Subsequent isolates of the same species from the same patient within a 14-day deduplication window, adapted from the CDC/NHSN Repeat Infection Timeframe (Centers for Disease Control and Prevention, 2026), were removed, retaining the first isolate.

### Laboratory methods

#### Organism classification and antimicrobial susceptibility testing

Organisms were grouped as Enterobacterales or non-fermenting GNB. Identification and AST were performed on the VITEK 2 system (bioMérieux, Marcy-l’Étoile, France); MALDI-TOF MS was unavailable. MICs were interpreted using Clinical and Laboratory Standards Institute (CLSI) breakpoints current at testing; CLSI M100 editions in use spanned the 23rd through 34th editions over the study period (2013–2024), and changes in interpretive criteria during this interval (notably for carbapenems among Enterobacterales and for fluoroquinolones) were applied prospectively as released, without retrospective re-interpretation. Susceptibility was dichotomized as susceptible vs non-susceptible (intermediate grouped with resistant). Intrinsic-resistance and nonreportable combinations were handled per CLSI-based rules (eMethods).

#### Non-susceptibility phenotype definitions

Fluoroquinolone (FQ-NS), extended-spectrum cephalosporin (ESC-NS), and carbapenem non-susceptibility were defined as non-susceptibility to at least one tested agent within the corresponding class.

DTR was defined for Enterobacterales, *Pseudomonas aeruginosa*, and *Acinetobacter baumannii* per Kadri et al. (2018) as non-susceptibility to all tested first-line agents across five categories: carbapenems (imipenem, meropenem, ertapenem), extended-spectrum cephalosporins (ceftriaxone, cefotaxime, ceftazidime, cefepime), fluoroquinolones (ciprofloxacin, levofloxacin), piperacillin-tazobactam, and aztreonam (or ampicillin-sulbactam for *Acinetobacter baumannii*); *Stenotrophomonas maltophilia* was excluded from DTR, the non-susceptibility hierarchy, and active-agent analyses.

### Outcomes

Co-primary outcomes were the annual proportion of Enterobacterales among respiratory GNB (and its temporal trend) and 30-day in-hospital mortality (death during the index admission within 30 days of respiratory culture collection) by organism–non-susceptibility composite category; patients discharged alive before day 30 were classified as survivors, and post-discharge deaths and readmissions were not captured because the study lacked linkage to vital statistics or post-discharge records. Secondary outcomes included organism- and species-level trends in FQ-NS, ESC-NS, carbapenem non-susceptibility, and DTR; mortality by non-susceptibility hierarchy; and a dose–response analysis of first-line active-agent availability.

Mortality was modeled using three exposure frameworks: a six-level organism–non-susceptibility composite, a five-level non-susceptibility hierarchy (Kadri et al., 2018), and a first-line active-agent count in three categories. Full definitions are in the eMethods.

### Statistical analysis

#### Descriptive statistics

Categorical variables were summarized as frequencies (%) and continuous variables as medians (IQR). Baseline and mortality analyses used the first respiratory GNB culture per admission to avoid patient-level duplication and bias from repeat cultures. Three analytic cohorts were used: the full isolate-episode cohort (N = 6,999) for temporal-trend analyses; the admission-level cohort (N = 3,946; one episode per admission) for baseline characterization; and complete-case analytic subsets within the admission cohort for the three mortality models (composite, N = 3,615; hierarchy, N = 3,890; dose-response, N = 3,392), with sample-size variation reflecting differences in exposure denominators (notably the exclusion of *Stenotrophomonas maltophilia* from DTR-based exposures) and complete-case requirements per model (**Supplementary eTable 15**).

#### Trend modeling

Trends were estimated using segmented (joinpoint) binomial regression with up to two joinpoints (Kim et al., 2000; Kim et al., 2023), yielding annual percent change (APC) and length-weighted average APC (AAPC) with delta-method 95% confidence intervals (CIs) (Clegg et al., 2009). Subgroup trend analyses by sex and ICU setting were performed when feasible. Full methodology, joinpoint selection, and feasibility criteria appear in the eMethods (**Supplementary eTable 14**).

#### Mortality analysis

Associations between non-susceptibility phenotypes and 30-day in-hospital mortality were estimated with modified Poisson regression. Models were adjusted for age, sex, ICU at culture, concordant bacteremia (± 7 days), healthcare-associated infection (culture >2 days after admission), COVID-19 era (2020–2024), calendar year, and the Elixhauser Comorbidity Index (Elixhauser et al., 1998; van Walraven et al., 2009). Continuous covariates were winsorized at the 1st and 99th percentiles and z-standardized; linearity testing and restricted-cubic-spline dose–response curves are reported in **Supplementary eTable 8** and **Supplementary eFigure 5** (**Supplementary eMethods**). A dose–response across first-line active categories was tested with an ordinal contrast; organism-group interaction was evaluated for the non-susceptibility hierarchy. Given multiple trend tests across organism groups and three exposure frameworks for mortality, primary inference was prespecified to focus on the DTR–mortality association in the organism–non-susceptibility composite model; remaining analyses were considered secondary or hypothesis-generating, and no formal correction for multiple comparisons was applied.

#### Sensitivity analyses

Six prespecified sensitivity analyses assessed robustness (overall in-hospital mortality, first admission per patient, ICU exclusion, concordant bacteremia exclusion, last vs first culture per admission, and staged regression to decompose confounding).

Complete susceptibility data were required per model; missing values were not imputed (**Supplementary eTable 15**). Tests were 2-sided with P<.05 considered significant. Analyses used Stata 19.5 and Python 3.12.

## Results

### Study population

**Figure 1:**
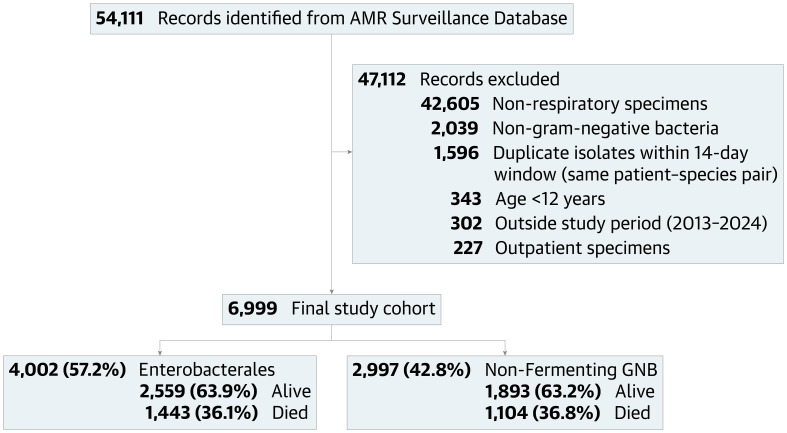
Study flow diagram. Of 54,111 isolates in the institutional surveillance database (2013–2024), 11,506 were respiratory specimens. After sequential exclusions the final cohort comprised 6,999 isolate-episodes from 3,808 unique patients (2013–2024). AMR, Antimicrobial Resistance; GNB, gram-negative bacilli.

Of 54,111 surveillance database records, 11,506 respiratory specimens remained after exclusions; sequential removal of non–gram-negative isolates, 14-day duplicates, patients <12 years, out-of-period records, and outpatient specimens yielded 6,999 isolate-episodes from 3,808 unique patients (**Figure 1**). Enterobacterales accounted for 57.2% (n=4,002) and non-fermenting GNB for 42.8% (n=2,997). Baseline characteristics of the admission-level cohort (N = 3,946 admissions from 3,808 patients) are in **Table 1**: median age 54 years (IQR, 30–72); 69.6% men; sputum (52.7%) and endotracheal aspirate (39.9%) were most common; 47.8% ICU-acquired; 74.2% healthcare-associated; median length of stay 27 days (IQR, 14–48); concordant bacteremia 7.8%.

**Table 1:** Baseline characteristics of respiratory gram-negative bacilli episodes (N = 3,946) ^a^.

Characteristic	Total (N = 3,946)	Organism group	
		Enterobacterales (n = 2,275)	Non-fermenting GNB (n = 1,671)
Demographics			
Age, yrs, median (IQR)	54 (30–72)	55 (33–72)	51 (26–73)
Category, n (%)			
Adolescent (12–17)	194 (4.9)	92 (4.0)	102 (6.1)
Adult (18–64)	2,285 (57.9)	1,331 (58.5)	954 (57.1)
Older (≥65)	1,467 (37.2)	852 (37.5)	615 (36.8)
Sex, n (%)			
Men	2,748 (69.6)	1,606 (70.6)	1,142 (68.3)
Women	1,198 (30.4)	669 (29.4)	529 (31.7)
Clinical Setting & Hospitalization			
Intensive care unit at culture, n (%)	1,885 (47.8)	1,033 (45.4)	852 (51.0)
Healthcare-associated (>2 days), n (%)	2,928 (74.2)	1,595 (70.1)	1,333 (79.8)
COVID-19 era (2020–2024), n (%)	1,706 (43.2)	1,121 (49.3)	585 (35.0)
Length of stay, days, median (IQR) ^b^	27 (14–48)	25 (13–45)	30 (16–54)
Days from admission to culture, median (IQR) ^c^	8 (2–17)	7 (2–17)	10 (4–18)
Concordant bacteremia, n (%) ^d^	306 (7.8)	203 (8.9)	103 (6.2)
Specimen Type			
Bronchial lavage	40 (1.0)	22 (1.0)	18 (1.1)
Endotracheal tube aspirate	1,575 (39.9)	875 (38.5)	700 (41.9)
Sputum	2,078 (52.7)	1,274 (56.0)	804 (48.1)
Tracheal secretion	253 (6.4)	104 (4.6)	149 (8.9)
Comorbidity Burden			
Autoimmune/connective tissue diseases, n (%)	128 (3.2)	72 (3.2)	56 (3.4)
Malignancy, n (%)	310 (7.9)	220 (9.7)	90 (5.4)
Chronic pulmonary disease, n (%)	160 (4.1)	82 (3.6)	78 (4.7)
Diabetes mellitus, n (%)	581 (14.7)	346 (15.2)	235 (14.1)
Hypertension, n (%)	1,417 (35.9)	842 (37.0)	575 (34.4)
Ischemic heart disease, n (%)	125 (3.2)	80 (3.5)	45 (2.7)
Renal failure ^e^, n (%)	255 (6.5)	145 (6.4)	110 (6.6)
Stroke, n (%)	470 (11.9)	296 (13.0)	174 (10.4)
Other comorbidities, n (%) ^f^	532 (13.5)	304 (13.4)	228 (13.6)
Elixhauser Comorbidity Index, median (IQR)	0 (0–3)	0 (0–3)	0 (0–0)

COVID-19, coronavirus disease 2019; ECI, Elixhauser Comorbidity Index; GNB, gram-negative bacilli; ICU, intensive care unit; IQR, interquartile range; N, total admissions; n, number with characteristic.

^a^Admission-level cohort: one culture per admission (first respiratory gram-negative bacilli [GNB] episode), yielding 3,946 admission records from 3,808 unique patients (drawn from 6,999 total isolate-episodes).

^b^Length of stay from admission date to discharge date.

^c^Days from admission to first culture collection.

^d^Same patient, same species blood culture within ±7 days of respiratory culture.

^e^Renal failure (Elixhauser Comorbidity Index [ECI] category) operationalized using three hospital-coded diagnoses: acute kidney injury, chronic kidney disease, and end-stage kidney disease. These diagnoses are listed separately in **Supplementary eTable 1** (marked ^b^) because they are not individual ECI categories; they served as source variables for the composite renal failure ECI component.

**f** Remaining 18 ECI categories not listed individually; see **Supplementary eTable 1** for full comorbidity detail.

Non-fermenting GNB episodes had a higher ICU proportion (51.0% vs 45.4%) and longer length of stay than Enterobacterales episodes; Enterobacterales episodes predominated during the COVID-19 era (49.3% vs 35.0%) (**Table 1**). Common comorbidities were hypertension (35.9%), diabetes (14.7%), and stroke (11.9%) (**Supplementary eTable 1**). Concordant bacteremia was associated with higher 30-day mortality (54.6% vs 25.7%; **Supplementary eTable 2**). The cohort comprised 68 species across 27 genera (**Supplementary eTable 3**), with an overall DTR prevalence of 37.6% (**Table 2**); in adjusted analyses, DTR was the phenotype most strongly associated with 30-day mortality (**Table 3**).

**Table 2:** Carbapenem non-susceptibility and difficult-to-treat proportions and temporal trends among respiratory gram-negative bacilli, 2013–2024.

Organism/group	N	N (%)	Joinpoints (Years)	APC segment 1% (95% CI)	APC segment 2% (95% CI)	APC segment 3% (95% CI)	AAPC % (95% CI)	Trend
Carbapenem non-susceptibility^c^								
All isolates	6,492	3,428 (52.8)	2022	7.1 (6.1–8.1)	−23.0 (−28.4 to −17.6)	—	0.9 (−0.4 to 2.2)	Stable
Enterobacterales	3,784	1,421 (37.6)	2016, 2021	−2.5 (−9.9 to 4.8)	28.4 (24.4–32.5)	−17.9 (−22.3 to −13.6)	5.4 (3.4–7.5)	Increasing
└─ *Klebsiella pneumoniae*	1,989	1,091 (54.9)	2020, 2022	20.9 (16.9–25.0)	0.9 (−4.0 to 5.8)	−23.6 (−31.2 to −15.9)	7.7 (5.0–10.3)	Increasing
Acinetobacter baumannii	1,503	1,386 (92.2)	2021	1.0 (0.5–1.5)	−7.9 (−11.3 to −4.5)	—	−1.5 (−2.5 to −0.5)	Decreasing
Pseudomonas aeruginosa	1,205	621 (51.5)	Linear	3.8 (2.2–5.4)	—	—	3.8 (2.2–5.4)	Increasing
Difficult-to-Treat resistance^d^								
All isolates	6,003	2,259 (37.6)	2015, 2022	30.8 (19.3–42.3)	5.5 (3.8–7.2)	−33.6 (−41.7 to −25.5)	0.8 (−1.7 to 3.4)	Stable
Enterobacterales	3,348	904 (27.0)	2021	29.1 (25.3–32.8)	−23.0 (−28.8 to −17.2)	—	12.1 (9.4–14.8)	Increasing
└─ *Klebsiella pneumoniae*	1,807	776 (42.9)	2021	23.8 (19.4–28.1)	−18.8 (−24.1 to −13.5)	—	10.3 (7.3–13.4)	Increasing
Acinetobacter baumannii	1,438	1,143 (79.5)	2015	12.7 (7.6–17.8)	−4.6 (−5.6 to −3.6)	—	−1.7 (−2.7 to −0.7)	Decreasing
Pseudomonas aeruginosa	1,147	208 (18.1)	Linear	4.9 (1.1–8.8)	—	—	4.9 (1.1–8.8)	Increasing

AAPC, average annual percent change; APC, annual percent change; CI, confidence interval.

^a^APC estimated using segmented binomial regression (log link) with joinpoints selected via weighted Bayesian Information Criterion (WBIC), enforcing ≥2-year spacing. AAPC represents weighted averages across segments; 95% CIs derived using the delta method.

^b^Trend defined as: increasing (CI entirely >0), decreasing (CI entirely <0), stable (CI crosses 0).

^c^Carbapenem non-susceptibility defined as non-susceptibility to ≥1 carbapenem:

Enterobacterales (imipenem, meropenem, ertapenem; imipenem excluded for Proteus spp. per CLSI),

*Pseudomonas aeruginosa* and *Acinetobacter baumannii* (imipenem or meropenem only).

Denominator N = 6,492.

^d^DTR defined per Kadri et al. (2018); restricted to Enterobacterales, *A. baumannii*, and *P. aeruginosa*.

*Stenotrophomonas maltophilia* excluded (N = 70; events = 4). Denominator N = 6,003.

**Table 3:** Antimicrobial non-susceptibility and 30-day mortality among respiratory gram-negative bacilli, 2013–2024 ^a^.

Variable	N	Died n (%)	aRR (95% CI)	*P* value
Primary outcome: Organism-Resistance Composite (N = 3,615)				
Enterobacterales				
Carbapenem-susceptible (ref.)	1342	271 (20.2)	1.00	—
Carbapenem-non-susceptible ^b^	240	58 (24.2)	1.00 (0.81 to 1.24)	0.972
Difficult-to-treat	562	253 (45.0)	1.34 (1.16 to 1.55)	<0.001
Non-fermenting GNB				
Carbapenem-susceptible	381	62 (16.3)	0.84 (0.67 to 1.06)	0.147
Carbapenem-non-susceptible ^b^	234	66 (28.2)	1.07 (0.87 to 1.33)	0.511
Difficult-to-treat	856	305 (35.6)	1.28 (1.12 to 1.46)	<0.001
Secondary outcomes				
Resistance Hierarchy ^c^ (N = 3,890)				
Susceptible (ref.)	1136	205 (18.0)	1.00	—
Fluoroquinolone-non-susceptible	297	71 (23.9)	1.20 (0.96 to 1.49)	0.103
Extended-spectrum cephalosporin-non-susceptible ^d^	477	109 (22.9)	1.09 (0.89 to 1.34)	0.391
Carbapenem-non-susceptible	562	144 (25.6)	1.11 (0.94 to 1.31)	0.236
Difficult-to-treat	1418	558 (39.4)	1.40 (1.22 to 1.60)	<0.001
Non-fermenting GNB (vs Enterobacterales)	1652	483 (29.2)	0.98 (0.88 to 1.08)	0.618
Dose-Response by Active Categories ^e^ (N = 3,392)				
Adequate (3–5 active; ref.)	1293	225 (17.4)	1.00	—
Limited (1–2 active)	683	187 (27.4)	1.22 (1.04 to 1.43)	0.014
Difficult-to-treat (0 active)	1416	558 (39.4)	1.47 (1.28 to 1.67)	<0.001
Non-fermenting GNB (vs Enterobacterales)	1455	431 (29.6)	0.95 (0.86 to 1.05)	0.300

aRR, adjusted risk ratio; CI, confidence interval; GNB, gram-negative bacilli.

**a** 30-day in-hospital mortality outcome; analysis restricted to the first culture per admission. Modified Poisson regression with robust standard errors clustered by patient was used. All models adjusted for age, sex, intensive care unit (ICU) admission at culture date, concordant bacteremia, healthcare-associated infection, COVID-19 era, calendar year, and Elixhauser Comorbidity Index (ECI; van Walraven weighting).

**b** The carbapenem-non-susceptible category excludes meeting criteria for difficult-to-treat (DTR).

**c** Non-susceptible classified using a 5-level mutually exclusive hierarchy: non-resistant, fluoroquinolone-resistant, extended-spectrum cephalosporin-resistant (ESC-NS), carbapenem-resistant, and DTR, as defined by Kadri et al. (2018).

**d** ESC-NS is defined only for Enterobacterales. Non-fermenting GNB bypasses the ESC-NS tier and are classified directly as non-resistant, fluoroquinolone-resistant, carbapenem-resistant, or DTR, as applicable.

**e** Five first-line antibiotic categories were evaluated for activity: (1) carbapenems (imipenem, meropenem, ertapenem), (2) extended-spectrum cephalosporins (ceftriaxone, cefotaxime, ceftazidime, cefepime), (3) fluoroquinolones (ciprofloxacin, levofloxacin), (4) piperacillin-tazobactam (when tested), and (5) aztreonam for non-*Acinetobacter* species or ampicillin-sulbactam for *Acinetobacter baumannii* (when tested). Analysis restricted to *Enterobacterales*, *Pseudomonas aeruginosa*, and *Acinetobacter baumannii* (reference group: ≥3 active categories; Kadri et al., 2018). *P*-for-trend from ordinal contrast across active-category groups (*P* < 0.001).

### Antimicrobial susceptibility

Non-fermenting GNB showed higher carbapenem non-susceptibility than Enterobacterales (imipenem 75.5% vs 33.9%; meropenem 71.0% vs 33.6%; both P<.001) and higher gentamicin and piperacillin-tazobactam non-susceptibility (all P<.001). Fluoroquinolone non-susceptibility was similar across groups (ciprofloxacin 68.5% vs 67.5%; P = .401) (**Supplementary eTables 4**, **5**; **Supplementary eFigure 1**).

### Temporal trends

#### Organism-level trends

**Figure 2:**
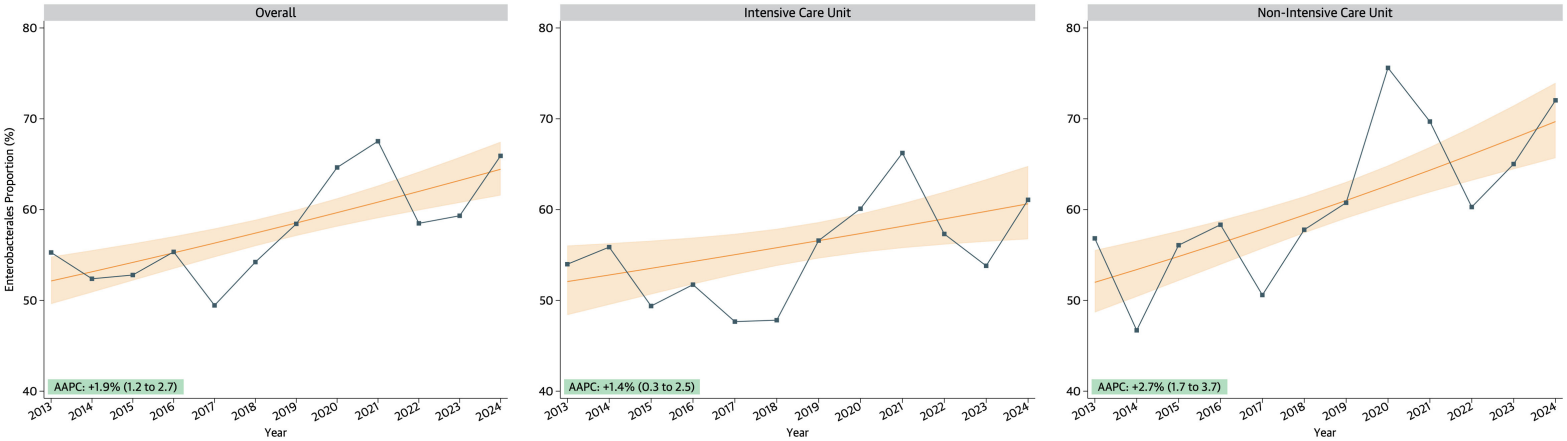
Temporal trends of Enterobacterales proportion among respiratory gram-negative bacilli. Observed proportions (connected markers) with segmented binomial regression trend lines and 95% confidence bands. Average annual percentage change (AAPC) with confidence intervals is annotated in each panel.

Enterobacterales increased steadily (AAPC, + 1.9%; 95% CI, 1.2 to 2.7) while non-fermenting GNB declined (AAPC, −2.5%; 95% CI, −3.5 to −1.6) (**Figure 2**; **Supplementary eTables 6, 7**). The non-fermenting decline was larger outside the ICU (ΔAAPC, 2.1 percentage points; 95% CI, 0.2 to 4.0; **Supplementary eTable 13**). Among Enterobacterales, *Klebsiella pneumoniae* predominated (29.1%; n=2,039); *Proteus mirabilis* and *Providencia stuartii* declined (AAPC, −18.2% and −22.0%), while *Enterobacter cloacae* increased (AAPC, + 9.0%). *Escherichia coli* and *Serratia marcescens* were stable. Among non-fermenters, *Acinetobacter baumannii* (21.9%) showed trend reversal in 2015 (overall AAPC, −0.7%); *Pseudomonas aeruginosa* declined linearly (AAPC, −4.5%) (**Supplementary eFigures 2, 3**).

#### Non-susceptibility phenotypes

Among Enterobacterales, FQ-NS (AAPC, −3.7%) and ESC-NS (AAPC, −4.5%) declined (**Supplementary eTable 6**). Overall carbapenem non-susceptibility was 52.8% and stable (AAPC, + 0.9%; **Table 2**; **Supplementary eFigure 4**). Within Enterobacterales, carbapenem non-susceptibility rose significantly (AAPC, + 5.4%), driven by *K. pneumoniae* (AAPC, + 7.7%). *A. baumannii* declined from 92.2% (AAPC, −1.5%), whereas carbapenem non-susceptibility in *P. aeruginosa* increased linearly (AAPC, + 3.8%).

DTR prevalence was 37.6% and stable overall (AAPC, + 0.8%; **Table 2**; **Supplementary eFigure 4**). Among Enterobacterales, DTR rose sharply (AAPC, + 12.1%), driven by *K. pneumoniae* (AAPC, + 10.3%). DTR *A. baumannii* declined (AAPC, −1.7%), while DTR *P. aeruginosa* increased (AAPC, + 4.9%).

### Mortality analysis

30-day in-hospital mortality was 27.9% (1,102/3,946 admissions) (**Supplementary eTable 2**).

#### Organism-non-susceptibility composite

In the fully adjusted composite model (N = 3,615), DTR was associated with increased 30-day mortality in both organism groups (**Table 3**). Relative to carbapenem-susceptible Enterobacterales, DTR Enterobacterales had an aRR of 1.34 (95% CI, 1.16–1.55) and DTR non-fermenting GNB aRR 1.28 (95% CI, 1.12–1.46). Carbapenem non-susceptibility without DTR was not associated with higher mortality (**Supplementary eTable 9**; **Supplementary eFigure 6**).

#### Non-susceptibility hierarchy

Using the 5-level hierarchy (N = 3,890), only DTR was associated with increased mortality (aRR, 1.40; 95% CI, 1.22–1.60); FQ-NS, ESC-NS, and carbapenem non-susceptibility were not (**Table 3**; **Supplementary eTable 10**). Organism group was not associated with mortality (aRR, 0.98) (**Table 3**), with no interaction (P = .447) (**Supplementary eTable 12**).

#### Dose-response

**Figure 3:**
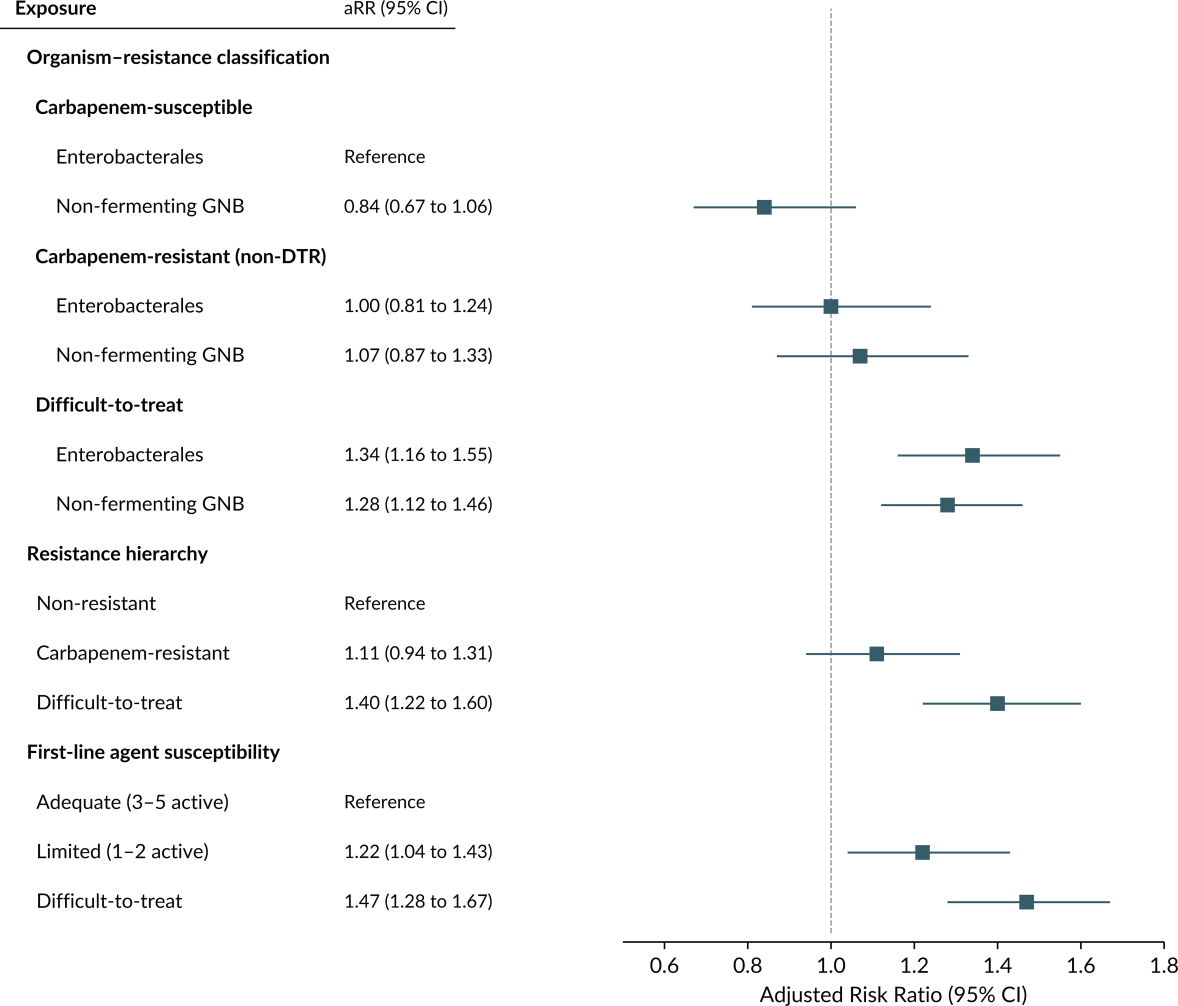
Adjusted risk ratios for 30-day mortality across 3 non-susceptibility exposure frameworks among respiratory gram-negative bacilli.

Mortality rose as active first-line agents decreased (P-trend<.001; N = 3,392): 1–2 active aRR 1.22 (95% CI, 1.04–1.43); no active agents (DTR) aRR 1.47 (95% CI, 1.28–1.67) (**Table 3**; **Supplementary eTable 11**; **Supplementary eFigure 7**). Consistency across all three frameworks is shown in **Figure 3**.

### Sensitivity analyses

In staged adjustment, the DTR–mortality association attenuated most after adding ICU status, consistent with partial severity-related confounding. Across six sensitivity specifications, DTR remained significantly associated with mortality in both organism groups (**Supplementary eTable 12**; **Supplementary eFigures 8, 9**).

## Discussion

### Epidemiologic shift and non-susceptibility trends

We observed a sustained shift in respiratory GNB epidemiology, with rising Enterobacterales and declining non-fermenting organisms across settings, though non-fermenters remained prevalent in the ICU. Among Enterobacterales, *Klebsiella pneumoniae* showed the most dynamic increase.

These findings align with regional GCC and Saudi Arabian data reporting GNB predominance in healthcare-associated respiratory infections and rising Enterobacterales resistance, particularly *K. pneumoniae* (El-Saed et al., 2016; Zowawi, 2016; Abdalla et al., 2023; Centers for Disease Control and Prevention, 2026; Shabi et al., 2026). The increasing Enterobacterales contribution likely reflects local differences in antimicrobial exposure, infection control, and case-mix.

Non-susceptibility patterns evolved heterogeneously. Carbapenem non-susceptibility rose among Enterobacterales, driven by *K. pneumoniae*, while FQ-NS and ESC-NS declined. Non-fermenting GNB showed stable or declining carbapenem non-susceptibility overall, with decreasing resistance in *A. baumannii* but increasing resistance in *P. aeruginosa*. Aggregate stability masked opposing organism-level trends, notably the DTR increase among Enterobacterales.

The carbapenem non-susceptibility increase is consistent with Saudi Arabian reports of expanding carbapenem-resistant *K. pneumoniae*, including OXA-48 producers (Zowawi, 2016; Alkhelaiwi et al., 2026; Shabi et al., 2026). The stable or declining non-fermenting resistance in our cohort differs from reports of persistently high ICU resistance (El-Saed et al., 2016; World Health Organization Regional Office for the Eastern Mediterranean, 2020; Abdalla et al., 2023), emphasizing the need for context-specific surveillance.

### Clinical impact of non-susceptibility phenotypes

Across all frameworks, DTR showed the strongest and most consistent adjusted association with 30-day mortality, whereas carbapenem non-susceptibility without DTR was not, indicating the limited prognostic value of single-class definitions.

These findings extend the work of Kadri et al. (2018), who reported that DTR outperformed conventional resistance classifications in stratifying outcomes in bloodstream infection, by demonstrating a similar adjusted association in respiratory gram-negative bacilli episodes and supporting the broader applicability of the framework.

The hierarchy analysis reinforces this: earlier phenotypes (FQ-NS, ESC-NS) were not associated with mortality in adjusted analyses, suggesting that clinical impact may relate to cumulative therapeutic limitation rather than non-susceptibility to any single class.

The dose–response analysis shows a graded mortality increase as active first-line options decline, with DTR (no active agents) carrying the highest risk, consistent with contemporary guidance framing DTR as a clinically meaningful decision-making construct (Kadri et al., 2018; Tamma et al., 2022).

Organism group was not associated with mortality in adjusted analyses and showed no interaction with non-susceptibility phenotype, suggesting that resistance phenotype, rather than pathogen identity, was the more relevant correlate of outcome in this cohort.

### Implications for practice and surveillance

These findings support non-susceptibility-centered frameworks in surveillance and clinical decision-making. Traditional metrics such as carbapenem non-susceptibility alone may underestimate risk by missing cumulative therapy loss, whereas DTR integrates microbiologic non-susceptibility with therapeutic consequence.

Early DTR identification could facilitate therapy optimization and risk stratification in critically ill patients; incorporating DTR into surveillance reporting may improve detection of clinically relevant patterns and inform empiric strategies.

### Limitations

This study has several limitations. First, its single-center retrospective design limits generalizability and introduces potential residual confounding and information bias. Second, the culture-based design without standardized clinical or radiographic adjudication (e.g., CDC/NHSN HAP/VAP criteria) cannot reliably distinguish true respiratory infection from airway colonization, particularly for sputum and endotracheal aspirate specimens; mortality associations should therefore be interpreted as reflecting culture-positive respiratory episodes rather than adjudicated pneumonia. Third, physiologic severity-of-illness measures (e.g., SOFA, APACHE II, vasopressor requirement, mechanical ventilation) and antimicrobial treatment data (timeliness, *in-vitro* appropriateness, source control) were unavailable; residual confounding by unmeasured severity, and the contribution of delayed or inappropriate empiric therapy, may therefore drive part of the observed mortality associations, which should be interpreted as associations of DTR with mortality rather than as evidence of an independent causal effect. Fourth, CLSI breakpoints evolved over the 12-year study period (M100 editions 23–34), with notable revisions for carbapenems among Enterobacterales and for fluoroquinolones; because raw MIC data were unavailable for retrospective re-interpretation, breakpoints were applied prospectively as released, so part of the observed temporal trends in carbapenem and fluoroquinolone non-susceptibility may reflect breakpoint evolution rather than true changes in pathogen susceptibility. Fifth, molecular characterization of non-susceptibility mechanisms was not consistently performed. Finally, outcomes were limited to in-hospital mortality, and longer-term outcomes such as post-discharge mortality and readmissions were not captured.

## Conclusion

In this large retrospective cohort of respiratory gram-negative bacilli episodes, we demonstrate an evolving epidemiology with increasing contribution of Enterobacterales alongside heterogeneous non-susceptibility trends. DTR, rather than carbapenem non-susceptibility alone or organism group, was consistently associated with 30-day mortality across all three exposure frameworks; given residual confounding by unmeasured illness severity and antimicrobial treatment, these findings should be interpreted as associations rather than evidence of an independent causal effect. These findings support a resistance-centered framework for surveillance and clinical risk stratification and highlight the importance of early identification of patients with limited therapeutic options.

## Data Availability

The raw data supporting the conclusions of this article will be made available by the authors, without undue reservation.
