# Supplementary material for: Difficult-to-treat resistance, not carbapenem non-susceptibility, is associated with 30-day mortality in respiratory gram-negative bacilli: a 12-year surveillance cohort
Source: Front Cell Infect Microbiol. 2026 Jul 20;16:1871310. doi: 10.3389/fcimb.2026.1871310 (PMC13429785; doi:10.3389/fcimb.2026.1871310)
Supplement: Supplementary file 1 [file DataSheet1.docx]

# **eMETHOD**

## Study Population

Patients <12 years were excluded because pediatric services were transferred from Aseer Central Hospital (ACH) to a dedicated pediatric hospital early in the study period; retaining these records would have introduced a structural artifact into temporal trend analyses.

Eligibility criteria required isolates to be recovered from respiratory specimens (sputum, endotracheal aspirate, tracheal secretions, or bronchoalveolar lavage) during the study period. The study was restricted to hospitalized patients; specimens collected in the emergency department were included only when collected during an encounter that resulted in hospital admission, and outpatient specimens were excluded. To preserve outcome independence, subsequent isolates of the same species from the same patient within a 14-day deduplication window, adapted from the CDC/NHSN Repeat Infection Timeframe, were excluded, with only the first isolate retained.

## Data Source and Extracted Variables

Culture-positive respiratory isolates and antimicrobial susceptibility testing (AST) results were extracted from the hospital microbiology database, which is part of an ongoing institutional antimicrobial resistance surveillance program. Extracted variables included patient demographics; specimen characteristics and culture data (organism identification and antimicrobial susceptibility profiles); and admission-related clinical information (ward location, comorbidities, length of stay, and disposition status). The study period extended from January 1, 2013 through August 27, 2024, yielding 12 annual time points; records were available through August 27, 2024 because the institutional transition to a new electronic health record platform precluded retrieval of later data. Because the investigation was designed as a culture-based surveillance study, analyses characterized organism distribution and antimicrobial resistance patterns among culture-positive isolates, and formal clinical or radiographic diagnostic criteria for respiratory infection (e.g., CDC/NHSN hospital-acquired pneumonia [HAP] or ventilator-associated pneumonia [VAP] definitions) were not applied.

## Microbiological Methods

This supplement expands the main laboratory methods by specifying the organism-reporting rules used for descriptive susceptibility analyses and phenotype derivation. Organisms were classified as Enterobacterales or non-fermenting gram-negative bacilli using taxonomic assignment and glucose fermentation status. Susceptibility results were harmonized to susceptible vs non-susceptible, with intermediate results grouped with resistant results to reduce misclassification from temporal breakpoint revisions. Organism-antibiotic pairs were excluded when the Clinical and Laboratory Standards Institute (CLSI) identified intrinsic resistance, when organism-specific interpretive criteria were unavailable, or when the pair was prespecified as nonreportable in the respiratory-study workflow [1].

Organism identification and AST were performed using the VITEK 2 automated system (bioMérieux, Marcy-l'Étoile, France) with gram-negative (GN) identification and gram-negative antimicrobial susceptibility testing (AST-GN) cards. Matrix-assisted laser desorption/ionization time-of-flight mass spectrometry (MALDI-TOF MS) was unavailable at the institution during the study period. Minimum inhibitory concentrations (MICs) were interpreted according to CLSI breakpoints current at the time of testing; CLSI M100 editions in use spanned the 23rd through 34th editions over the study period (2013–2024), and changes in interpretive criteria during this interval (notably for carbapenems among Enterobacterales and for fluoroquinolones) were applied prospectively as released, without retrospective re-interpretation; raw MIC values were unavailable for retrospective reinterpretation.

Moxifloxacin was excluded from organism-group comparisons and fluoroquinolone phenotype derivation because CLSI M100 does not provide interpretive breakpoints across the non-fermenting gram-negative bacilli spectrum used in those analyses. For non-fermenting gram-negative bacilli, first-generation cephalosporins, second-generation cephalosporins, and cephamycins were treated as nonreportable. Additional species-specific exclusions followed CLSI M100, Appendix B.

## Resistance Phenotype Definitions

Fluoroquinolone resistance was defined for all gram-negative bacilli as non-susceptibility to ciprofloxacin or levofloxacin. Extended-spectrum cephalosporin resistance was defined for Enterobacterales only as non-susceptibility to cefepime, ceftazidime, cefotaxime, or ceftriaxone. Carbapenem-resistant Enterobacterales were defined as Enterobacterales non-susceptible to at least 1 carbapenem. For non-Proteeae Enterobacterales, carbapenems included imipenem, meropenem, and ertapenem. For Proteeae (*Proteus*, *Providencia*, and *Morganella*), carbapenems included meropenem and ertapenem only because elevated imipenem minimum inhibitory concentrations (MICs) may reflect non-carbapenemase mechanisms under CLSI footnote d [1]. For non-fermenting gram-negative bacilli, carbapenem resistance in *Acinetobacter baumannii* and *Pseudomonas aeruginosa* was defined as non-susceptibility to imipenem or meropenem, with ertapenem not applicable [1].

The mutually exclusive resistance hierarchy comprised non-resistant, fluoroquinolone-NS (FQ-NS), extended-spectrum cephalosporin-resistant (ESC-NS), carbapenem-non-susceptible, and difficult-to-treat resistant (DTR) categories. The 6-level organism-non-susceptible composite combined organism group with carbapenem non-susceptible and DTR status. DTR was defined using organism-specific criteria adapted from Kadri et al [2] and was evaluated only when at least 1 carbapenem, 1 extended-spectrum cephalosporin, and 1 fluoroquinolone had been tested. For Enterobacterales, DTR required non-susceptibility to all tested carbapenems, all tested extended-spectrum cephalosporins, and all tested fluoroquinolones, with piperacillin-tazobactam and aztreonam contributing when tested. For Protease, the carbapenem component used meropenem and ertapenem only. For *P. aeruginosa*, DTR required non-susceptibility to imipenem and meropenem, ceftazidime and cefepime, ciprofloxacin and levofloxacin, and to piperacillin-tazobactam and aztreonam when tested. For *A. baumannii*, DTR required non-susceptibility to imipenem and meropenem, extended-spectrum cephalosporins, ciprofloxacin and levofloxacin, and to ampicillin-sulbactam and piperacillin-tazobactam when tested.

*Stenotrophomonas maltophilia* was excluded from primary DTR, the resistance hierarchy, and first-line active-agent derivation because the original Kadri framework did not include this organism and the standard 5-category first-line structure was not applicable. *Burkholderia cepacia* complex (n = 32) was also excluded from standard phenotype derivation because the available CLSI breakpoint framework was insufficient for routine breakpoint-based classification in this study workflow. The first-line active-agent count used the same organism-specific category set as the DTR definition and was grouped as 3 to 5 active categories, 1 to 2 active categories, or 0 active categories.

## Statistical Methods

### Outcomes and Descriptive Statistics

Co-primary outcomes were the annual proportion of Enterobacterales among respiratory GNB isolates (and its temporal trend), and 30-day in-hospital mortality, defined as death during the index admission within 30 days of respiratory culture collection, by organism–non-susceptibility composite category. Secondary outcomes included organism-level proportion trends; organism-specific temporal changes in FQ-NS, ESC-NS, carbapenem non-susceptibility, and DTR; mortality associations across the non-susceptibility hierarchy; and a dose–response analysis of first-line antimicrobial category availability in relation to 30-day mortality.

Categorical variables were summarized as frequencies (percentages) and continuous variables as medians with interquartile ranges (IQR). Baseline characteristics and mortality analyses were restricted to the first respiratory GNB culture recorded during each admission to avoid counting the same patient more than once and to reduce bias from subsequent cultures that may reflect colonization or treatment-related effects.

### Analytic Cohorts

Three analytic cohorts were used. The full isolate-episode cohort (N = 6,999) supported all temporal-trend analyses, including organism-level proportions, species-level proportions, and resistance phenotype trends. The admission-level cohort (N = 3,946; one episode per admission, derived from 3,808 unique patients) supported baseline characterization and was the basis for all mortality analyses. Within the admission cohort, three complete-case analytic subsets were used for the three mortality models: the organism–non-susceptibility composite model (N = 3,615), the resistance hierarchy model (N = 3,890), and the dose–response model based on first-line active-agent count (N = 3,392). Sample-size variation across these mortality subsets reflects two structural features. First, exposure denominators differ across frameworks because *Stenotrophomonas maltophilia* and *Burkholderia cepacia* complex are excluded from DTR-based exposures. Second, complete-case requirements differ across frameworks because models require complete antimicrobial susceptibility data for the categories that define each exposure (e.g., the dose–response model requires testing in all five first-line categories used to compute the active-agent count). Variable-level completeness by organism group is reported in eTable 15.

### Segmented Binomial Regression Model

The annual probability of each binary outcome (organism proportions, species proportions, and resistance phenotypes) for isolate $i$ in calendar year $t$ was modeled using a piecewise log-linear formulation with a binomial distribution and log link:

$$loglog \left( p_{t} \right) = \beta_{0}+\beta_{1}t+\sum_{j=1}^{k} \delta_{j}\left( t-\tau_{j} \right)$$

where $p_{it}$ is the probability of the outcome, $\beta_{1}$ is the baseline slope (log risk ratio per year), $\kappa$ is the number of joinpoints (0, 1, or 2), $\tauⱼ$ are the joinpoint locations (calendar years), $\deltaⱼ$ are the change-in-slope parameters at each joinpoint, and $\left( t - \tauⱼ \right)^{+}=\left( t - \tauⱼ, 0 \right)$ is the positive-part function ensuring slope changes apply only beyond the joinpoint. The within-segment slope for segment $s$ is $\beta^{1}+ \Sigma_{\left\{ j\leq s \right\}}\deltaⱼ$, from which the annual percentage change (APC) is derived as:

$$APC=100\left( e^{\beta_{s}}-1 \right)\%$$

Over a user-specified interval $\left[ a, b \right]$, the average APC (AAPC) is the length-weighted average of segment slopes (1):

$$AAPC=100\left( exp\left( \sum_{s} w_{s}\beta_{s} \right)-1 \right)\%, where w_{s}=\frac{years in \left[ a, b \right] \cap segments s}{b-a}$$

Confidence intervals for APC and AAPC were derived using the delta method [3]. For the difference in AAPC (ΔAAPC) between 2 independent strata, analytic confidence intervals used the National Cancer Institute (NCI) Joinpoint variance formulation [4].

### Jointpoint Selection

Candidate models with $\kappa$ = 0, 1, or 2 joinpoints were fit via grid search over allowable year positions, constrained to a minimum of 2 years from study boundaries (2013, 2024) and 2 years between breakpoints. The final $\kappa$ was selected using the weighted Bayesian information criterion (WBIC) described by Kim et al [5], with comparability considerations informed by prior segmented-regression work from the same group [6]. Patient-clustered robust standard errors were used throughout to account for multiple isolates per person.

### Covariate Preprocessing and Staged Mortality Modeling

Continuous covariables (age and Elixhauser Comorbidity Index) were winsorized at the 1st and 99th percentiles and z-standardized (mean = 0, standard deviation [SD] = 1). Linearity was assessed by comparing linear, restricted cubic spline (RCS), and fractional polynomial (FP) specifications using Akaike information criterion (AIC) differences greater than 2 and Wald tests (*P* < .05). Age and calendar year were modeled as RCS with 3 knots, and the Elixhauser index was retained as linear. To decompose confounding pathways, staged regression added covariable blocks sequentially: stage 1 included demographics (age and sex), comorbidity (Elixhauser Comorbidity Index), healthcare-associated infection, COVID-19 era, and calendar year; stage 2 added intensive care unit (ICU) status at culture; and stage 3 added concordant bacteremia. The primary organism-resistance composite model had an AIC of 3,892.9.

Mortality models were adjusted for age, sex, ICU at culture, concordant bacteremia (blood culture positive for the same species within ±7 days), healthcare-associated infection (culture >2 days after admission), COVID-19 era (2020–2024 vs 2013–2019), calendar year, and the Elixhauser Comorbidity Index. A dose–response relationship across first-line active categories was evaluated using an ordinal contrast (P value for trend). To determine whether the association between resistance and mortality differed by organism group, we fit a non-susceptibility hierarchy × organism group interaction model.

### Sensitivity Analyses

Six prespecified sensitivity analyses assessed robustness under alternative specifications: (1) overall in-hospital mortality as the outcome; (2) restriction to the first admission per patient; (3) exclusion of ICU from covariables; (4) exclusion of concordant bacteremia from covariables; (5) use of the last (vs first) culture per admission; and (6) staged regression to decompose confounding pathways. Model-specific complete susceptibility data were required, and missing values were not imputed.

### Multiple Comparisons

The analytic plan involved multiple trend tests across organism groups and species, and three exposure frameworks for the mortality outcome (organism–non-susceptibility composite, resistance hierarchy, and dose–response across first-line active categories). Primary inference was prespecified to focus on the DTR–mortality association in the organism–non-susceptibility composite model, which is the model most directly aligned with the primary clinical question. The remaining trend, hierarchy, and dose–response analyses were considered secondary and complementary, intended to triangulate the primary finding under alternative parameterizations rather than to test independent hypotheses. Sensitivity analyses (described above) were considered confirmatory of the primary inference under alternative model specifications. Given this hierarchical inferential structure, no formal correction for multiple comparisons (e.g., Bonferroni or Benjamini-Hochberg adjustment) was applied to nominal P values; instead, consistency of effect direction and magnitude across frameworks was used as the principal criterion for inferential robustness, supplemented by the convergence of point estimates and confidence intervals across the prespecified sensitivity specifications.

### Subgroup Trend Eligibility

Subgroup trend analyses by sex (men vs women) and by ICU setting (ICU vs non-ICU) were conducted for species proportions and resistance phenotypes. Each stratification was gated independently, so an outcome could qualify for the sex comparison but not the ICU comparison, or vice versa. Trend analyses were restricted to outcomes meeting prespecified feasibility criteria: at least 50 events, at least 100 evaluable isolates, and an outcome prevalence between 0.5% and 99.5%. These thresholds were informed by simulation evidence showing that logistic-family models require approximately 10 to 20 events per parameter to avoid bias [7, 8]. Because each segmented regression model contained 2 to 4 parameters, 50 events provided an events-per-parameter ratio of at least 12.5, exceeding the minimum recommended by Vittinghoff and McCulloch [8]. The ICU stratum comprised 3,778 isolates (54.0% of the isolate-level cohort). The complete feasibility assessment, including event counts per stratum and inclusion or exclusion decisions for each outcome, is reported in eTable 14.

Species-specific resistance phenotypes (carbapenem-resistant *K. pneumoniae*, *E. coli*, *A. baumannii*, and *P. aeruginosa*; difficult-to-treat resistant Enterobacterales, *K. pneumoniae*, *E. coli*, *A. baumannii*, and *P. aeruginosa*) were assessed independently for each comparison; most were excluded from 1 or both subgroup comparisons because the organism-specific denominator, when further split by sex or ICU setting, fell below the 50-event threshold in at least 1 stratum. For each included outcome, stratum-specific breakpoints were selected independently using the same WBIC procedure, allowing different joinpoint structures across strata. ΔAAPC was computed by subtracting women from men and non-ICU from ICU, with analytic confidence intervals obtained from the same NCI Joinpoint variance formulation used for the main trend models [4].

# **eRESULTS**

## Subgroup Trends by Sex and ICU Setting

No significant sex-based differences were observed for Enterobacterales or non-fermenting GNB proportions. Among individual species, *Acinetobacter baumannii* showed the clearest divergence by care setting: its proportion was stable in ICU settings but declined significantly in non-ICU wards. *Klebsiella pneumoniae* increased in both settings but more rapidly in ICU settings. *Pseudomonas aeruginosa* declined uniformly across men, women, ICU, and non-ICU settings, with no significant difference in either stratification. No significant sex-based differences were observed for any species proportion.

For non-susceptibility phenotypes, carbapenem non-susceptibility among Enterobacterales was the only phenotype showing a significant sex-based difference, rising substantially faster in men than women. Carbapenem non-susceptibility increased significantly faster in ICU settings than non-ICU settings, as did carbapenem non-susceptibility among Enterobacterales. DTR increased in ICU settings but declined in non-ICU settings.

# **eREFERENCES**

1. Clinical and Laboratory Standards Institute (CLSI). Performance standards for antimicrobial susceptibility testing. 36th ed. CLSI supplement M100. Wayne (PA): CLSI; 2026.
2. Kadri SS, Adjemian J, Lai YL, Spaulding AB, Ricotta E, Prevots DR, et al. Difficult-to-treat resistance in gram-negative bacteremia at 173 US hospitals: retrospective cohort analysis of prevalence, predictors, and outcome of resistance to all first-line agents. Clin Infect Dis. 2018;67(12):1803–14. doi:10.1093/cid/ciy378.
3. Clegg LX, Hankey BF, Tiwari R, Feuer EJ, Edwards BK. Estimating average annual per cent change in trend analysis. Stat Med. 2009;28(28):3670–3682. doi:10.1002/sim.3733.
4. National Cancer Institute. Joinpoint Regression Program, version 5.2.0. Statistical Research and Applications Branch, Surveillance Research Program. Bethesda (MD): National Cancer Institute; 2024.
5. Kim HJ, Chen HS, Midthune D, Wheeler B, Buckman DW, Green D, et al. Data-driven choice of a model selection method in joinpoint regression. J Appl Stat. 2023;50(9):1992–2013. doi:10.1080/02664763.2022.2063265.
6. Kim HJ, Fay MP, Yu B, Barrett MJ, Feuer EJ. Comparability of segmented line regression models. Biometrics. 2004;60(4):1005–1014. doi:10.1111/j.0006-341X.2004.00256.x.
7. Peduzzi P, Concato J, Kemper E, Holford TR, Feinstein AR. A simulation study of the number of events per variable in logistic regression analysis. J Clin Epidemiol. 1996;49(12):1373–1379. doi:10.1016/S0895-4356(96)00236-3.
8. Vittinghoff E, McCulloch CE. Relaxing the rule of ten events per variable in logistic and Cox regression. Am J Epidemiol. 2007;165(6):710–718. doi:10.1093/aje/kwk052.
