# Supplementary material for: Difficult-to-treat resistance, not carbapenem non-susceptibility, is associated with 30-day mortality in respiratory gram-negative bacilli: a 12-year surveillance cohort
Source: Front Cell Infect Microbiol. 2026 Jul 20;16:1871310. doi: 10.3389/fcimb.2026.1871310 (PMC13429785; doi:10.3389/fcimb.2026.1871310)
Supplement: Supplementary file 2 [file Table1.docx]

**Supplementary Materials**

**eTable 1.** Full Comorbidity Profile by Organism Group Among Respiratory Gram-Negative Bacilli (N = 3,946) ᵃ

| **Comorbidity** | **Total (N = 3,946)** | **Organism Group** | |
| --- | --- | --- | --- |
|  |  | **Enterobacterales (n = 2,275)** | **Non-fermenting GNB (n = 1,671)** |
| Acute kidney injury ᵇ | 64 (1.6) | 34 (1.5) | 30 (1.8) |
| Asthma ᵇ | 56 (1.4) | 26 (1.1) | 30 (1.8) |
| Atrial fibrillation ᵇ | 33 (0.8) | 22 (1.0) | 11 (0.7) |
| Autoimmune/connective tissue diseases | 128 (3.2) | 72 (3.2) | 56 (3.4) |
| Cardiac arrhythmias | 39 (1.0) | 27 (1.2) | 12 (0.7) |
| Chronic kidney disease ᵇ | 207 (5.2) | 117 (5.1) | 90 (5.4) |
| Chronic liver disease | 28 (0.7) | 18 (0.8) | 10 (0.6) |
| Chronic obstructive pulmonary disease ᵇ | 57 (1.4) | 37 (1.6) | 20 (1.2) |
| Chronic pulmonary disease | 160 (4.1) | 82 (3.6) | 78 (4.7) |
| Coagulopathy | 1 (0.0) | 1 (0.0) | 0 (0.0) |
| Congestive heart failure | 74 (1.9) | 39 (1.7) | 35 (2.1) |
| Deep vein thrombosis ᵇ | 37 (0.9) | 20 (0.9) | 17 (1.0) |
| Deficiency anemia | 16 (0.4) | 8 (0.4) | 8 (0.5) |
| Dementia ᵇ | 54 (1.4) | 35 (1.5) | 19 (1.1) |
| Depression | 11 (0.3) | 7 (0.3) | 4 (0.2) |
| Diabetes mellitus | 581 (14.7) | 346 (15.2) | 235 (14.1) |
| Complicated | 112 (2.8) | 67 (2.9) | 45 (2.7) |
| Uncomplicated | 469 (11.9) | 279 (12.3) | 190 (11.4) |
| End-stage kidney disease ᵇ | 142 (3.6) | 80 (3.5) | 62 (3.7) |
| Epilepsy ᵇ | 75 (1.9) | 44 (1.9) | 31 (1.9) |
| Fluid/electrolyte disorders | 17 (0.4) | 12 (0.5) | 5 (0.3) |
| HIV/AIDS | 5 (0.1) | 3 (0.1) | 2 (0.1) |
| Hypertension | 1417 (35.9) | 842 (37.0) | 575 (34.4) |
| Complicated | 371 (9.4) | 221 (9.7) | 150 (9.0) |
| Uncomplicated | 1046 (26.5) | 621 (27.3) | 425 (25.4) |
| Hypothyroidism | 92 (2.3) | 55 (2.4) | 37 (2.2) |
| Ischemic heart disease ᵇ | 125 (3.2) | 80 (3.5) | 45 (2.7) |
| Malignancy (any) | 310 (7.9) | 220 (9.7) | 90 (5.4) |
| Lymphoma/leukemia | 40 (1.0) | 29 (1.3) | 11 (0.7) |
| Metastatic cancer | 19 (0.5) | 13 (0.6) | 6 (0.4) |
| Solid tumor without metastasis | 251 (6.4) | 178 (7.8) | 73 (4.4) |
| Myocardial infarction ᵇ | 70 (1.8) | 46 (2.0) | 24 (1.4) |
| Obesity | 17 (0.4) | 6 (0.3) | 11 (0.7) |
| Other neurological disorders | 121 (3.1) | 64 (2.8) | 57 (3.4) |
| Paralysis | 27 (0.7) | 7 (0.3) | 20 (1.2) |
| Peptic ulcer disease | 3 (0.1) | 0 (0.0) | 3 (0.2) |
| Peripheral vascular disease | 39 (1.0) | 28 (1.2) | 11 (0.7) |
| Psychoses | 19 (0.5) | 12 (0.5) | 7 (0.4) |
| Pulmonary circulation disorders | 71 (1.8) | 40 (1.8) | 31 (1.9) |
| Pulmonary embolism ᵇ | 70 (1.8) | 40 (1.8) | 30 (1.8) |
| Renal failure | 255 (6.5) | 145 (6.4) | 110 (6.6) |
| Rheumatoid arthritis ᵇ | 2 (0.1) | 1 (0.0) | 1 (0.1) |
| Sickle cell disease ᵇ | 21 (0.5) | 12 (0.5) | 9 (0.5) |
| Stroke ᵇ | 470 (11.9) | 296 (13.0) | 174 (10.4) |
| Systemic lupus erythematosus ᵇ | 32 (0.8) | 17 (0.7) | 15 (0.9) |
| Valvular heart disease | 4 (0.1) | 3 (0.1) | 1 (0.1) |
| Weight loss | 3 (0.1) | 3 (0.1) | 0 (0.0) |
| Abbreviations: AIDS, acquired immunodeficiency syndrome; GNB, gram-negative bacilli; HIV, human immunodeficiency virus; N, total admissions; n, number with comorbidity. | | | |
| a—Restricted to the first respiratory gram-negative bacilli culture per hospital admission (same cohort as Table 1).  b—Not included in the standard Elixhauser Comorbidity Index. | | | |

**eTable 2.** Characteristics of Respiratory Gram-Negative Bacilli Episodes by Concordant Bacteremia Status (N = 3,946) ᵃ

| **Characteristic** | **Total**  **(N = 3,946)** | **Concordant Bacteremia Status ᵇ** | |
| --- | --- | --- | --- |
|  |  | **No Concordant Bacteremia**  **(n = 3,640)** | **Concordant Bacteremia**  **(n = 306)** |
| Demographics | | | |
| Age, yrs, median (IQR) | 54 (30–72) | 54 (29–72) | 59 (39–75) |
| Women, n (%) | 1,198 (30.4) | 1,103 (30.3) | 95 (31.0) |
| Organism Group | | | |
| Non-fermenting GNB, n (%) | 1,671 (42.3) | 1,568 (43.1) | 103 (33.7) |
| Top Species | | | |
| *Klebsiella pneumoniae*, n (%) | 1,399 (35.5) | 1,219 (33.5) | 180 (58.8) |
| *Acinetobacter baumannii*, n (%) | 1,016 (25.7) | 927 (25.5) | 89 (29.1) |
| *Pseudomonas aeruginosa*, n (%) | 509 (12.9) | 497 (13.7) | 12 (3.9) |
| *Proteus mirabilis*, n (%) | 105 (2.7) | 102 (2.8) | 3 (1.0) |
| *Escherichia coli*, n (%) | 338 (8.6) | 328 (9.0) | 10 (3.3) |
| Clinical Setting | | | |
| Intensive care unit at culture, n (%) | 1,885 (47.8) | 1,653 (45.4) | 232 (75.8) |
| Healthcare-associated (>2 days), n (%) | 2,928 (74.2) | 2,652 (72.9) | 276 (90.2) |
| Length of stay, days, median (IQR) | 27 (14–48) | 27 (14–49) | 29 (17–44) |
| Days from admission to culture, median (IQR) | 8 (2–17) | 8 (2–17) | 12 (7–20) |
| Non-susceptible Phenotype ᶜ | | | |
| Extended-spectrum cephalosporin-non-susceptible, n/N (%) | 1,254/2,275 (55.1) | 1,085/2,072 (52.4) | 169/203 (83.3) |
| Carbapenem-non-susceptible Enterobacterales, n/N (%) | 857/2,275 (37.7) | 700/2,072 (33.8) | 157/203 (77.3) |
| Carbapenem-non-susceptible (Overall), n/N (%) | 1,978/3,701 (53.4) | 1,728/3,401 (50.8) | 250/300 (83.3) |
| Difficult-to-treat (Overall), n/N (%) | 1,418/3,444 (41.2) | 1,212/3,158 (38.4) | 206/286 (72.0) |
| COVID-19 era | | | |
| COVID-19 era (2020–2024), n (%) | 1,706 (43.2) | 1,516 (41.6) | 190 (62.1) |
| Comorbidity Burden | | | |
| Elixhauser Comorbidity Index, median (IQR) | 0 (0–3) | 0 (0–3) | 0 (0–0) |
| Outcome | | | |
| 30-day mortality, n (%) | 1,102 (27.9) | 935 (25.7) | 167 (54.6) |
| BSI Timing (among concordant BSI) | | | |
| Blood culture before respiratory (-7 to -1 days) | — | — | 71 (23.2) |
| Same day (0 days) | — | — | 152 (49.7) |
| Blood culture after respiratory (+1 to +7 days) | — | — | 83 (27.1) |
| Abbreviations: BSI, bloodstream infection; COVID, coronavirus disease 2019; GNB, gram-negative bacilli; IQR, interquartile range; n, number with characteristic; N, number evaluable. | | | |
| a—Admission-level cohort: one culture per admission (first respiratory gram-negative bacilli [GNB] episode), yielding 3,946 admission records from 3,808 unique patients (drawn from 6,999 total isolate-episodes).  b—Concordant bloodstream infection (BSI): same patient, same species blood culture within ±7 days of respiratory culture.  c—Non-susceptible denominators vary by phenotype: Carbapenem-non-susceptible Enterobacterales and Extended-spectrum cephalosporin-non-susceptible are restricted to Enterobacterales only; other phenotypes include all GNB. | | | |

**eTable 3.** Full Species Inventory by Year Among Respiratory Gram-Negative Bacilli, 2013–2024 (N = 6,999)

| **Species** | **2013** | **2014** | **2015** | **2016** | **2017** | **2018** | **2019** | **2020** | **2021** | **2022** | **2023** | **2024** | **Total** | **%** |
| --- | --- | --- | --- | --- | --- | --- | --- | --- | --- | --- | --- | --- | --- | --- |
| Enterobacterales | | | | | | | | | | | | | | |
| *Cedecea davisae* | 1 | 1 | — | — | — | — | — | — | — | — | — | — | 2 | 0.0 |
| *Cedecea lapagei* | 1 | — | — | — | — | — | — | — | — | — | — | — | 1 | 0.0 |
| *Cedecea sp.* | — | — | 1 | — | — | — | — | — | — | — | — | — | 1 | 0.0 |
| *Citrobacter amalonaticus* | — | — | — | — | 1 | — | — | — | — | — | — | — | 1 | 0.0 |
| *Citrobacter braakii* | 2 | 1 | — | — | — | — | — | — | — | — | — | — | 3 | 0.0 |
| *Citrobacter farmeri* | 1 | 1 | — | — | — | — | — | — | — | — | — | 1 | 3 | 0.0 |
| *Citrobacter freundii* | 4 | 3 | 2 | 4 | 1 | — | — | — | 2 | — | 2 | — | 18 | 0.3 |
| *Citrobacter koseri* | 1 | 2 | — | 1 | 1 | 1 | — | — | — | — | 1 | — | 7 | 0.1 |
| *Citrobacter sp.* | — | 3 | 1 | — | — | — | 4 | 2 | 1 | — | — | — | 11 | 0.2 |
| *Citrobacter youngae* | — | — | — | — | — | — | — | — | — | — | — | 1 | 1 | 0.0 |
| *Cronobacter sakazakii* | — | 1 | — | — | — | — | — | — | — | — | — | — | 1 | 0.0 |
| *Enterobacter asburiae* | — | — | — | — | — | — | — | — | — | — | 1 | — | 1 | 0.0 |
| *Enterobacter cancerogenus* | 1 | — | — | — | — | — | — | — | 1 | — | — | — | 2 | 0.0 |
| *Enterobacter cloacae* | 17 | 15 | 9 | 8 | 15 | 12 | 15 | 12 | 23 | 16 | 20 | 8 | 170 | 2.4 |
| *Enterobacter hormaechei* | — | — | — | — | 1 | — | — | — | — | — | — | — | 1 | 0.0 |
| *Enterobacter sp.* | 1 | 1 | — | 2 | 2 | — | — | 1 | — | — | — | — | 7 | 0.1 |
| *Escherichia coli* | 63 | 47 | 45 | 57 | 40 | 51 | 33 | 25 | 32 | 23 | 44 | 21 | 481 | 6.9 |
| *Klebsiella aerogenes* | 16 | 10 | 16 | 5 | 7 | 6 | 12 | 11 | 9 | 5 | 11 | 9 | 117 | 1.7 |
| *Klebsiella ornithinolytica* | — | 2 | — | — | — | — | — | — | — | — | — | — | 2 | 0.0 |
| *Klebsiella oxytoca* | 4 | 3 | 2 | 1 | — | 1 | 7 | 4 | 1 | 4 | 8 | 10 | 45 | 0.6 |
| *Klebsiella pneumoniae* | 199 | 96 | 109 | 123 | 112 | 118 | 218 | 264 | 317 | 222 | 134 | 113 | 2,025 | 28.9 |
| *Klebsiella pneumoniae subsp. ozaenae* | — | — | 2 | — | 1 | 2 | 1 | 1 | 2 | 1 | 1 | 2 | 13 | 0.2 |
| *Klebsiella pneumoniae subsp. rhinoscleromatis* | — | — | — | — | — | — | — | — | — | — | — | 1 | 1 | 0.0 |
| *Klebsiella sp.* | 3 | 3 | 1 | — | 1 | — | — | — | 1 | 1 | 3 | — | 13 | 0.2 |
| *Kluyvera ascorbata* | — | — | — | — | 2 | — | — | — | — | — | — | — | 2 | 0.0 |
| *Kluyvera intermedia* | — | 1 | — | 1 | — | — | — | — | — | — | — | — | 2 | 0.0 |
| *Leclercia adecarboxylata* | — | — | — | — | — | — | — | — | — | — | — | 1 | 1 | 0.0 |
| *Lelliottia amnigena* | — | — | — | 1 | — | — | — | — | — | — | — | — | 1 | 0.0 |
| *Morganella morganii* | 13 | 35 | 21 | 11 | 5 | 2 | 1 | 2 | 2 | 3 | — | 1 | 96 | 1.4 |
| *Pantoea agglomerans* | — | 1 | 1 | 1 | 2 | — | — | — | — | — | 1 | — | 6 | 0.1 |
| *Pluralibacter gergoviae* | 1 | — | — | — | 2 | — | — | — | — | — | — | 1 | 4 | 0.1 |
| *Proteus mirabilis* | 94 | 90 | 76 | 112 | 68 | 39 | 29 | 22 | 13 | 18 | 8 | 1 | 570 | 8.1 |
| *Proteus penneri* | 2 | 4 | — | — | — | — | — | — | — | — | — | — | 6 | 0.1 |
| *Proteus sp.* | 10 | 10 | 4 | — | — | — | — | — | — | — | — | — | 24 | 0.3 |
| *Proteus vulgaris* | — | 1 | 1 | — | — | — | — | — | — | — | — | — | 2 | 0.0 |
| *Providencia alcalifaciens* | — | — | — | — | — | — | 1 | — | — | — | — | — | 1 | 0.0 |
| *Providencia rettgeri* | 2 | 1 | — | — | — | — | — | — | — | — | — | 1 | 4 | 0.1 |
| *Providencia rustigianii* | 1 | — | 1 | — | — | — | — | — | — | — | — | — | 2 | 0.0 |
| *Providencia sp.* | 6 | 2 | — | 1 | — | — | — | — | — | — | — | — | 9 | 0.1 |
| *Providencia stuartii* | 42 | 69 | 43 | 11 | 4 | 10 | 15 | 5 | 4 | 8 | 1 | — | 212 | 3.0 |
| *Serratia ficaria* | 1 | — | — | — | 1 | — | — | — | — | — | 1 | — | 3 | 0.0 |
| *Serratia liquefaciens* | 2 | 2 | 1 | 1 | — | — | — | — | — | — | — | — | 6 | 0.1 |
| *Serratia marcescens* | 18 | 8 | 11 | 12 | 6 | 2 | 14 | 13 | 8 | 9 | 8 | 4 | 113 | 1.6 |
| *Serratia odorifera* | — | 1 | — | — | — | — | — | — | — | — | — | 1 | 2 | 0.0 |
| *Serratia plymuthica* | 1 | — | 1 | — | — | — | — | — | — | — | — | — | 2 | 0.0 |
| *Serratia sp.* | 1 | 1 | 1 | — | — | — | — | — | — | — | 1 | — | 4 | 0.1 |
| *Tatumella ptyseos* | — | 2 | — | — | — | — | — | — | — | — | — | — | 2 | 0.0 |
| *Yersinia enterocolitica* | — | 1 | — | — | — | — | — | — | — | — | — | — | 1 | 0.0 |
| Subtotal | 508 | 418 | 349 | 352 | 272 | 244 | 350 | 362 | 416 | 310 | 245 | 176 | 4,002 | 57.2 |
| Non-fermenting GNB | | | | | | | | | | | | | | |
| *Achromobacter sp.* | 1 | 2 | — | — | — | — | — | — | — | — | — | 2 | 5 | 0.1 |
| *Acinetobacter baumannii* | 157 | 194 | 183 | 144 | 152 | 109 | 145 | 123 | 95 | 122 | 72 | 34 | 1,530 | 21.9 |
| *Acinetobacter calcoaceticus* | — | 2 | 3 | — | — | — | — | — | — | — | — | — | 5 | 0.1 |
| *Acinetobacter haemolyticus* | — | 3 | 2 | — | — | — | — | — | — | 1 | — | — | 6 | 0.1 |
| *Acinetobacter lwoffii* | 1 | 2 | 2 | 1 | — | 1 | — | — | 4 | — | 2 | 1 | 14 | 0.2 |
| *Acinetobacter sp.* | 5 | 3 | 2 | 1 | 2 | — | — | — | — | — | — | — | 13 | 0.2 |
| *Alcaligenes faecalis* | — | 1 | — | — | — | — | — | — | — | — | — | — | 1 | 0.0 |
| *Alcaligenes sp.* | — | 1 | — | — | — | — | — | — | — | — | — | — | 1 | 0.0 |
| *Burkholderia cepacia* | 4 | 2 | — | 5 | — | — | — | 8 | 7 | 1 | 1 | 3 | 31 | 0.4 |
| *Burkholderia sp.* | — | — | 1 | — | — | — | — | — | — | — | — | — | 1 | 0.0 |
| *Comamonas testosteroni* | — | — | — | — | 1 | — | — | — | — | — | — | — | 1 | 0.0 |
| *Moraxella catarrhalis* | — | — | — | — | — | — | — | — | 1 | — | — | — | 1 | 0.0 |
| *Moraxella sp.* | — | 1 | — | — | — | — | 1 | — | — | — | — | — | 2 | 0.0 |
| *Pseudomonas aeruginosa* | 221 | 156 | 107 | 122 | 109 | 87 | 92 | 53 | 73 | 89 | 79 | 36 | 1,224 | 17.5 |
| *Pseudomonas fluorescens* | 3 | 2 | — | 3 | 1 | 1 | 1 | — | — | — | — | — | 11 | 0.2 |
| *Pseudomonas luteola* | — | — | 1 | — | — | — | — | — | — | — | 1 | 1 | 3 | 0.0 |
| *Pseudomonas sp.* | 12 | 9 | 6 | 2 | 7 | 4 | 2 | 2 | 4 | 2 | 8 | 9 | 67 | 1.0 |
| *Ralstonia pickettii* | — | — | — | — | — | — | — | — | — | — | — | 1 | 1 | 0.0 |
| *Stenotrophomonas maltophilia* | 7 | 2 | 3 | 6 | 5 | 4 | 8 | 12 | 15 | 5 | 5 | 4 | 76 | 1.1 |
| *Stutzerimonas stutzeri* | — | — | 2 | — | 1 | — | — | — | 1 | — | — | — | 4 | 0.1 |
| Subtotal | 411 | 380 | 312 | 284 | 278 | 206 | 249 | 198 | 200 | 220 | 168 | 91 | 2,997 | 42.8 |
| Total | 919 | 798 | 661 | 636 | 550 | 450 | 599 | 560 | 616 | 530 | 413 | 267 | 6,999 | 100.0 |
| Abbreviations: GNB, gram-negative bacilli. | | | | | | | | | | | | | | |

**eTable 4.** Antimicrobial Non-Susceptibility Rates by Organism Group Among Respiratory Gram-Negative Bacilli (N = 6,999) ᵃ

| **Antibiotic** | **Total n/N (%)** | **Organism Group** | | **P value** |
| --- | --- | --- | --- | --- |
|  |  | **Enterobacterales n/N (%)** | **Non-fermenting GNB n/N (%)** |  |
| Aminoglycosides | | | | |
| Amikacin | 2453/4929 (49.8) | 1708/3807 (44.9) | 745/1122 (66.4) | <0.001 |
| Gentamicin | 3018/5176 (58.3) | 1837/3607 (50.9) | 1181/1569 (75.3) | <0.001 |
| Tobramycin | 2122/4056 (52.3) | 1246/2222 (56.1) | 876/1834 (47.8) | <0.001 |
| Carbapenems | | | | |
| Ertapenem | 996/2210 (45.1) | 996/2210 (45.1) | — | — |
| Imipenem | 3230/6263 (51.6) | 1221/3602 (33.9) | 2009/2661 (75.5) | <0.001 |
| Meropenem | 2954/6041 (48.9) | 1197/3567 (33.6) | 1757/2474 (71.0) | <0.001 |
| Cephalosporins | | | | |
| Cefazolin | 160/461 (34.7) | 160/461 (34.7) | — | — |
| Cefepime | 3945/5860 (67.3) | 2124/3262 (65.1) | 1821/2598 (70.1) | <0.001 |
| Cefotaxime | 1402/1918 (73.1) | 1011/1493 (67.7) | 391/425 (92.0) | <0.001 |
| Cefoxitin | 921/1804 (51.1) | 921/1804 (51.1) | — | — |
| Ceftazidime | 4021/5940 (67.7) | 2169/3250 (66.7) | 1852/2690 (68.8) | 0.084 |
| Ceftriaxone | 592/768 (77.1) | 412/582 (70.8) | 180/186 (96.8) | <0.001 |
| Cefuroxime | 1000/1387 (72.1) | 1000/1387 (72.1) | — | — |
| Fluoroquinolones | | | | |
| Ciprofloxacin | 4371/6436 (67.9) | 2538/3760 (67.5) | 1833/2676 (68.5) | 0.401 |
| Levofloxacin | 3160/4983 (63.4) | 1811/2885 (62.8) | 1349/2098 (64.3) | 0.271 |
| Penicillin + β-Lactamase Inhibitor | | | | |
| Amoxicillin-clavulanate | 1465/2412 (60.7) | 1465/2412 (60.7) | — | — |
| Ampicillin-sulbactam | 1676/2152 (77.9) | 1134/1523 (74.5) | 542/629 (86.2) | <0.001 |
| Piperacillin-tazobactam | 2461/5118 (48.1) | 1514/3499 (43.3) | 947/1619 (58.5) | <0.001 |
| Tetracyclines | | | | |
| Minocycline | 287/510 (56.3) | 197/348 (56.6) | 90/162 (55.6) | 0.848 |
| Tigecycline | 493/2275 (21.7) | 493/2274 (21.7) | — | — |
| Other | | | | |
| Ampicillin | 480/528 (90.9) | 480/528 (90.9) | — | — |
| Aztreonam | 1369/2184 (62.7) | 1101/1705 (64.6) | 268/479 (55.9) | <0.001 |
| Trimethoprim-sulfamethoxazole | 3061/5369 (57.0) | 2191/3734 (58.7) | 870/1635 (53.2) | <0.001 |
| Abbreviations: GNB, gram-negative bacilli; n, number non-susceptible; N, number tested. | | | | |
| a—Denominators vary by antibiotic: species-antibiotic combinations were excluded when Clinical and Laboratory Standards Institute (CLSI) interpretive criteria were not reportable for that organism or when combinations were suppressed by CLSI-informed project rules (CLSI M100-Ed36 Appendix B). | | | | |

**eTable 5.** Species-Specific Antimicrobial Non-Susceptibility Rates Among Respiratory Gram-Negative Bacilli ᵃ

| **Antibiotic** | **Enterobacterales** | | | | |  | **Non-fermenting GNB** | | |
| --- | --- | --- | --- | --- | --- | --- | --- | --- | --- |
|  | ***E. cloacae*** | ***E. coli*** | ***K. pneumoniae*** | ***P. mirabilis*** | ***P. stuartii*** |  | ***A. baumannii*** | ***P. aeruginosa*** | ***S. maltophilia*** |
| Aminoglycosides | | | | | | | | | |
| Amikacin | 27/165 (16.4) | 85/458 (18.6) | 972/1944 (50.0) | 389/534 (72.8) | 71/205 (34.6) |  | 709/997 (71.1) | NR | NR |
| Gentamicin | 43/167 (25.7) | 130/464 (28.0) | 964/1939 (49.7) | 489/540 (90.6) | NR |  | 1133/1451 (78.1) | NR | NR |
| Tobramycin | 28/112 (25.0) | 103/281 (36.7) | 793/1295 (61.2) | 210/241 (87.1) | NR |  | 631/972 (64.9) | 226/780 (29.0) | NR |
| Carbapenems | | | | | | | | | |
| Ertapenem | 33/109 (30.3) | 33/244 (13.5) | 831/1304 (63.7) | 18/207 (8.7) | 23/92 (25.0) |  | NR | NR | NR |
| Imipenem | 32/162 (19.8) | 38/442 (8.6) | 862/1913 (45.1) | 112/442 (25.3) | 71/189 (37.6) |  | 1352/1405 (96.2) | 593/1138 (52.1) | NR |
| Meropenem | 22/156 (14.1) | 38/445 (8.5) | 988/1850 (53.4) | 36/486 (7.4) | 35/179 (19.6) |  | 1219/1333 (91.4) | 489/1039 (47.1) | NR |
| Cephalosporins | | | | | | | | | |
| Cefepime | 51/148 (34.5) | 228/344 (66.3) | 1160/1726 (67.2) | 333/415 (80.2) | 143/184 (77.7) |  | 1287/1394 (92.3) | 471/1086 (43.4) | NR |
| Ceftriaxone | 8/23 (34.8) | 54/74 (73.0) | 231/319 (72.4) | 58/74 (78.4) | 14/16 (87.5) |  | 175/179 (97.8) | NR | NR |
| Cefotaxime | 46/83 (55.4) | 62/110 (56.4) | 585/878 (66.6) | 82/107 (76.6) | 110/117 (94.0) |  | 355/377 (94.2) | NR | NR |
| Ceftazidime | 75/152 (49.3) | 209/327 (63.9) | 1121/1702 (65.9) | 360/425 (84.7) | 164/196 (83.7) |  | 1302/1426 (91.3) | 489/1142 (42.8) | NR |
| Cefuroxime | 60/78 (76.9) | 71/122 (58.2) | 663/921 (72.0) | 76/97 (78.4) | 62/67 (92.5) |  | NR | NR | NR |
| Cefazolin | NR | 31/65 (47.7) | 78/317 (24.6) | 45/57 (78.9) | NR |  | NR | NR | NR |
| Cefoxitin | NR | 50/237 (21.1) | 736/1147 (64.2) | 59/229 (25.8) | 33/86 (38.4) |  | NR | NR | NR |
| Fluoroquinolones | | | | | | | | | |
| Ciprofloxacin | 60/164 (36.6) | 313/454 (68.9) | 1230/1929 (63.8) | 494/536 (92.2) | 182/195 (93.3) |  | 1295/1411 (91.8) | 469/1139 (41.2) | NR |
| Levofloxacin | 31/128 (24.2) | 245/367 (66.8) | 947/1513 (62.6) | 291/345 (84.3) | 138/154 (89.6) |  | 952/1099 (86.6) | 344/839 (41.0) | 17/70 (24.3) |
| Penicillin + β-Lactamase Inhibitor | | | | | | | | | |
| Amoxicillin-clavulanate | NR | 203/364 (55.8) | 1031/1562 (66.0) | 169/374 (45.2) | NR |  | NR | NR | NR |
| Ampicillin-sulbactam | NR | 139/200 (69.5) | 810/1096 (73.9) | 124/142 (87.3) | 38/39 (97.4) |  | 539/622 (86.7) | NR | NR |
| Piperacillin-tazobactam | 51/155 (32.9) | 108/442 (24.4) | 1103/1837 (60.0) | 61/468 (13.0) | 48/176 (27.3) |  | 513/562 (91.3) | 398/971 (41.0) | NR |
| Tetracyclines | | | | | | | | | |
| Minocycline | 6/22 (27.3) | 11/45 (24.4) | 174/260 (66.9) | NR | NR |  | 89/159 (56.0) | NR | NT |
| Tigecycline | 24/124 (19.4) | 12/334 (3.6) | 408/1555 (26.2) | NR | NR |  | NR | NR | NR |
| Other | | | | | | | | | |
| Ampicillin | NR | 190/221 (86.0) | NR | 263/274 (96.0) | NR |  | NR | NR | NR |
| Aztreonam | 59/95 (62.1) | 73/132 (55.3) | 750/1078 (69.6) | 76/155 (49.0) | 54/69 (78.3) |  | NR | 241/443 (54.4) | NR |
| Trimethoprim-sulfamethoxazole | 52/165 (31.5) | 210/460 (45.7) | 1084/1940 (55.9) | 456/498 (91.6) | 174/196 (88.8) |  | 801/1459 (54.9) | NR | 8/74 (10.8) |
| Abbreviations: GNB, gram-negative bacilli; NR, not reported (no validated Clinical and Laboratory Standards Institute [CLSI] interpretive criteria for that species-antibiotic combination or suppressed by CLSI-informed project rules [CLSI M100-Ed36 Appendix B]); NT, not tested (antibiotic not performed by the laboratory for this species). Species: *A. baumannii*, *Acinetobacter baumannii*; *E. cloacae*, *Enterobacter cloacae*; *E. coli*, *Escherichia coli*; *K. pneumoniae*, *Klebsiella pneumoniae*; *P. aeruginosa*, *Pseudomonas aeruginosa*; *P. mirabilis*, *Proteus mirabilis*; *P. stuartii*, *Providencia stuartii*; *S. maltophilia*, *Stenotrophomonas maltophilia*. | | | | | | | | | |
| a—Format: number non-susceptible / number tested (%).  NR = not reported — = not applicable | | | | | | | | | |


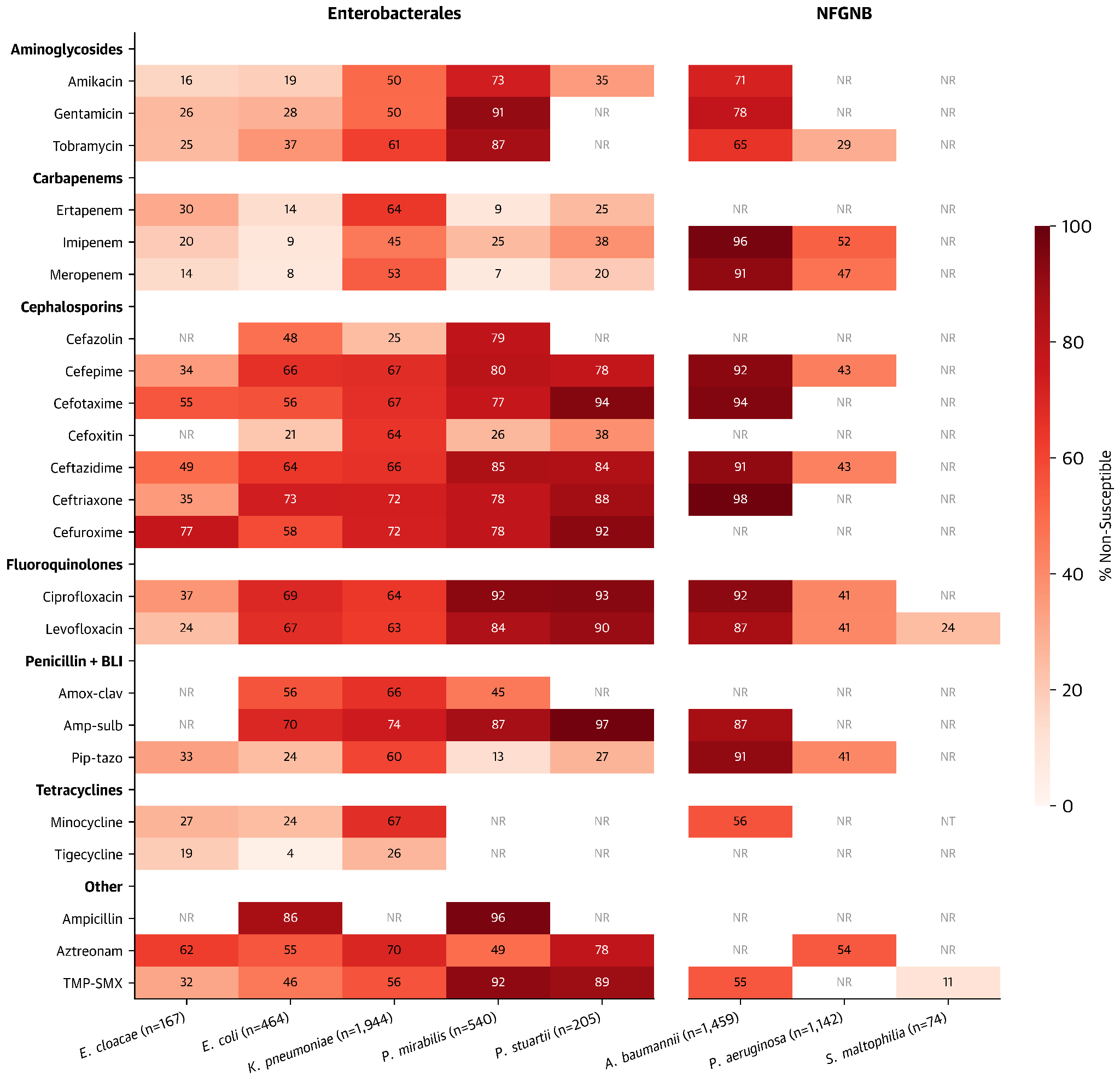
 **eFigure 1.** Antimicrobial Non-Susceptibility Rates Across Species and Drug Classes Among Respiratory Gram-Negative Bacilli. Cell values represent the percentage of non-susceptible isolates. NR, no validated Clinical and Laboratory Standards Institute (CLSI) breakpoint or intrinsic resistance; NT, not tested. Abbreviations: Amox-clav, amoxicillin-clavulanate; Amp-sulb, ampicillin-sulbactam; BLI, beta-lactamase inhibitor; NFGNB, non-fermenting gram-negative bacilli; Pip-tazo, piperacillin-tazobactam; TMP-SMX, trimethoprim-sulfamethoxazole. *A. baumannii*, *Acinetobacter baumannii*; *E. cloacae*, *Enterobacter cloacae*; *E. coli*, *Escherichia coli*; *K. pneumoniae*, *Klebsiella pneumoniae*; *P. aeruginosa*, *Pseudomonas aeruginosa*; *P. mirabilis*, *Proteus mirabilis*; *P. stuartii*, *Providencia stuartii*; *S. maltophilia*, *Stenotrophomonas maltophilia*.

**eTable 6.** Segmented Trend Model Selection and Average Annual Percentage Change (AAPC) Summary, 2013–2024

| **Outcome** | **Breakpoint(s) ᵃ** | **Segments** | **WBIC ᵃ** | **AAPC (%/yr) ᵃ** | **95% CI** | **Trend ᵇ** |
| --- | --- | --- | --- | --- | --- | --- |
| Enterobacterales proportion | | | | | | |
| Overall | Linear | 1 | 9553.0 | 1.9 | 1.2 to 2.7 | Increasing |
| ICU | Linear | 1 | 5209.9 | 1.4 | 0.3 to 2.5 | Increasing |
| Non-ICU | Linear | 1 | 4358.4 | 2.7 | 1.7 to 3.7 | Increasing |
| Non-fermenting GNB proportion | | | | | | |
| Overall | Linear | 1 | 9554.0 | -2.5 | -3.5 to -1.6 | Decreasing |
| ICU | Linear | 1 | 5210.2 | -1.7 | -3.0 to -0.4 | Decreasing |
| Non-ICU | Linear | 1 | 4359.5 | -3.8 | -5.1 to -2.4 | Decreasing |
| Species proportions | | | | | | |
| Enterobacterales | | | | | | |
| *Enterobacter cloacae* | Linear | 1 | 1620.3 | 9.0 | 4.1 to 14.0 | Increasing |
| *Escherichia coli* | Linear | 1 | 3539.4 | -0.4 | -4.1 to 3.3 | Stable |
| *Klebsiella aerogenes* | Linear | 1 | 1223.5 | 3.2 | -3.1 to 9.5 | Stable |
| *Klebsiella pneumoniae* | 2015, 2020 | 3 | 7992.9 | 6.0 | 4.3 to 7.7 | Increasing |
| *Morganella morganii* | Linear | 1 | 993.8 | -23.2 | -28.9 to -17.4 | Decreasing |
| *Proteus mirabilis* | 2016 | 2 | 3771.1 | -18.2 | -23.0 to -13.4 | Decreasing |
| *Providencia stuartii* | Linear | 1 | 1820.0 | -22.0 | -26.2 to -17.9 | Decreasing |
| *Serratia marcescens* | Linear | 1 | 1191.9 | 1.2 | -4.6 to 6.9 | Stable |
| Enterobacterales subtotal | Linear | 1 | 9553.0 | 1.9 | 1.2 to 2.7 | Increasing |
| Non-fermenting GNB | | | | | | |
| *Acinetobacter baumannii* | 2015 | 2 | 7354.0 | -0.7 | -2.4 to 0.9 | Stable |
| *Pseudomonas aeruginosa* | Linear | 1 | 6489.6 | -4.5 | -6.4 to -2.6 | Decreasing |
| *Stenotrophomonas maltophilia* | Linear | 1 | 862.6 | 12.1 | 5.2 to 19.0 | Increasing |
| Non-fermenting GNB subtotal | Linear | 1 | 9554.0 | -2.5 | -3.5 to -1.6 | Decreasing |
| Resistance phenotypes | | | | | | |
| Fluoroquinolone-resistance | 2019, 2021 | 3 | 4739.6 | -3.7 | -5.3 to -2.1 | Decreasing |
| Extended-spectrum cephalosporin-resistance | 2019, 2021 | 3 | 4223.2 | -4.5 | -5.7 to -3.4 | Decreasing |
| Carbapenem-resistant isolates | | | | | | |
| All isolates | 2022 | 2 | 8752.1 | 0.9 | -0.4 to 2.2 | Stable |
| Enterobacterales | 2016, 2021 | 3 | 4587.9 | 5.4 | 3.4 to 7.5 | Increasing |
| *Acinetobacter baumannii* | 2021 | 2 | 833.3 | -1.5 | -2.5 to -0.5 | Decreasing |
| *Escherichia coli* | Linear | 1 | 1515.7 | 5.0 | -8.0 to 18.0 | Stable |
| *Klebsiella pneumoniae* | 2020, 2022 | 3 | 2469.4 | 7.7 | 5.0 to 10.3 | Increasing |
| *Pseudomonas aeruginosa* | Linear | 1 | 1673.7 | 3.8 | 2.2 to 5.4 | Increasing |
| Difficult-to-treat (all evaluable) | | | | | | |
| All isolates | 2015, 2022 | 3 | 7779.8 | 0.8 | -1.7 to 3.4 | Stable |
| Enterobacterales | 2021 | 2 | 3502.9 | 12.1 | 9.4 to 14.8 | Increasing |
| *Acinetobacter baumannii* | 2015 | 2 | 1417.1 | -1.7 | -2.7 to -0.7 | Decreasing |
| *Escherichia coli* | Linear | 1 | 382.7 | 25.3 | -15.1 to 65.6 | Stable |
| *Klebsiella pneumoniae* | 2021 | 2 | 2238.1 | 10.3 | 7.3 to 13.4 | Increasing |
| *Pseudomonas aeruginosa* | Linear | 1 | 1106.9 | 4.9 | 1.1 to 8.8 | Increasing |
| Abbreviations: AAPC, average annual percentage change; CI, confidence interval; GNB, gram-negative bacilli; ICU, intensive care unit; WBIC, weighted Bayesian Information Criterion. | | | | | | |
| a—Annual percentage change (APC) from segmented binomial regression (log link) with data-driven breakpoints selected by weighted Bayesian Information Criterion (WBIC; Kim et al., 2023); minimum 2 years from study boundaries and 2 years between breakpoints. Grid search over 0-, 1-, and 2-breakpoint configurations; WBIC adapts penalty between Bayesian information criterion (BIC) and BIC₃ (penalty = 3k·ln[n]) based on maximum segment-specific pseudo-R². Average annual percentage change (AAPC) weighted across segments. 95% delta-method confidence intervals (CIs) (Clegg et al., 2009).  b—Trend direction: increasing (CI entirely above 0), decreasing (CI entirely below 0), stable (CI crosses 0). | | | | | | |

**eTable 7.** Species Proportions and Temporal Trends Among Respiratory Gram-Negative Bacilli, 2013–2024

| **Organism** | **n (%)** | **Breakpoint** |  | **Annual Percentage Change, % (95% CI) ᵃ** | | |  | **AAPC, % (95% CI) ᵃ** | **Trend ᵇ** |
| --- | --- | --- | --- | --- | --- | --- | --- | --- | --- |
|  |  |  |  | Segment 1 | Segment 2 | Segment 3 |  |  |  |
| Enterobacterales | | | | | | | | | |
| Overall | 4,002 (57.2) | Linear |  | 1.9 (1.2 to 2.7) | — | — |  | 1.9 (1.2 to 2.7) | Increasing |
| ICU | 2,105 (55.7) | Linear |  | 1.4 (0.3 to 2.5) | — | — |  | 1.4 (0.3 to 2.5) | Increasing |
| Non-ICU | 1,897 (58.9) | Linear |  | 2.7 (1.7 to 3.7) | — | — |  | 2.7 (1.7 to 3.7) | Increasing |
| Delta (ICU vs Non-ICU) ᶜ | — | — |  | — | — | — |  | -1.3 (-2.8 to 0.2) | Stable |
| Non-fermenting GNB | | | | | | | | | |
| Overall | 2,997 (42.8) | Linear |  | -2.5 (-3.5 to -1.6) | — | — |  | -2.5 (-3.5 to -1.6) | Decreasing |
| ICU | 1,673 (44.3) | Linear |  | -1.7 (-3.0 to -0.4) | — | — |  | -1.7 (-3.0 to -0.4) | Decreasing |
| Non-ICU | 1,324 (41.1) | Linear |  | -3.8 (-5.1 to -2.4) | — | — |  | -3.8 (-5.1 to -2.4) | Decreasing |
| Delta (ICU vs Non-ICU) ᶜ | — | — |  | — | — | — |  | 2.1 (0.2 to 4.0) | Increasing |
| Species Distribution | | | | | | | | | |
| Enterobacterales | | | | | | | | | |
| *Enterobacter cloacae* | 170 (2.4) | Linear |  | 9.0 (4.1 to 14.0) | — | — |  | 9.0 (4.1 to 14.0) | Increasing |
| *Escherichia coli* | 481 (6.9) | Linear |  | -0.4 (-4.1 to 3.3) | — | — |  | -0.4 (-4.1 to 3.3) | Stable |
| *Klebsiella aerogenes* | 117 (1.7) | Linear |  | 3.2 (-3.1 to 9.5) | — | — |  | 3.2 (-3.1 to 9.5) | Stable |
| *Klebsiella pneumoniae* | 2,039 (29.1) | 2015, 2020 |  | -17.7 (-26.5 to -8.9) | 29.3 (24.9 to 33.8) | -6.2 (-9.6 to -2.9) |  | 6.0 (4.3 to 7.7) | Increasing |
| *Morganella morganii* | 96 (1.4) | Linear |  | -23.2 (-28.9 to -17.4) | — | — |  | -23.2 (-28.9 to -17.4) | Decreasing |
| *Proteus mirabilis* | 570 (8.1) | 2016 |  | 19.3 (5.3 to 33.3) | -29.0 (-35.5 to -22.4) | — |  | -18.2 (-23.0 to -13.4) | Decreasing |
| *Providencia stuartii* | 212 (3.0) | Linear |  | -22.0 (-26.2 to -17.9) | — | — |  | -22.0 (-26.2 to -17.9) | Decreasing |
| *Serratia marcescens* | 113 (1.6) | Linear |  | 1.2 (-4.6 to 6.9) | — | — |  | 1.2 (-4.6 to 6.9) | Stable |
| Subtotal | 4,002 (57.2) | Linear |  | 1.9 (1.2 to 2.7) | — | — |  | 1.9 (1.2 to 2.7) | Increasing |
| Non-fermenting GNB | | | | | | | | | |
| *Acinetobacter baumannii* | 1,530 (21.9) | 2015 |  | 25.3 (14.8 to 35.7) | -5.7 (-7.6 to -3.8) | — |  | -0.7 (-2.4 to 0.9) | Stable |
| *Pseudomonas aeruginosa* | 1,224 (17.5) | Linear |  | -4.5 (-6.4 to -2.6) | — | — |  | -4.5 (-6.4 to -2.6) | Decreasing |
| *Stenotrophomonas maltophilia* | 76 (1.1) | Linear |  | 12.1 (5.2 to 19.0) | — | — |  | 12.1 (5.2 to 19.0) | Increasing |
| Subtotal | 2,997 (42.8) | Linear |  | -2.5 (-3.5 to -1.6) | — | — |  | -2.5 (-3.5 to -1.6) | Decreasing |
| Abbreviations: AAPC, average annual percentage change; CI, confidence interval; GNB, gram-negative bacilli; ICU, intensive care unit. | | | | | | | | | |
| a—Annual percentage change (APC) from segmented binomial regression (log link) with data-driven breakpoints selected by weighted Bayesian Information Criterion (WBIC; Kim et al., 2023); minimum 2 years from study boundaries and 2 years between breakpoints. Average annual percentage change (AAPC) weighted across segments. 95% delta-method confidence intervals (CIs) (Clegg et al., 2009).  b—Trend direction: increasing (CI entirely above 0), decreasing (CI entirely below 0), stable (CI crosses 0).  c—Delta: difference in AAPC between intensive care unit (ICU) and non-ICU settings (ICU minus non-ICU). | | | | | | | | | |


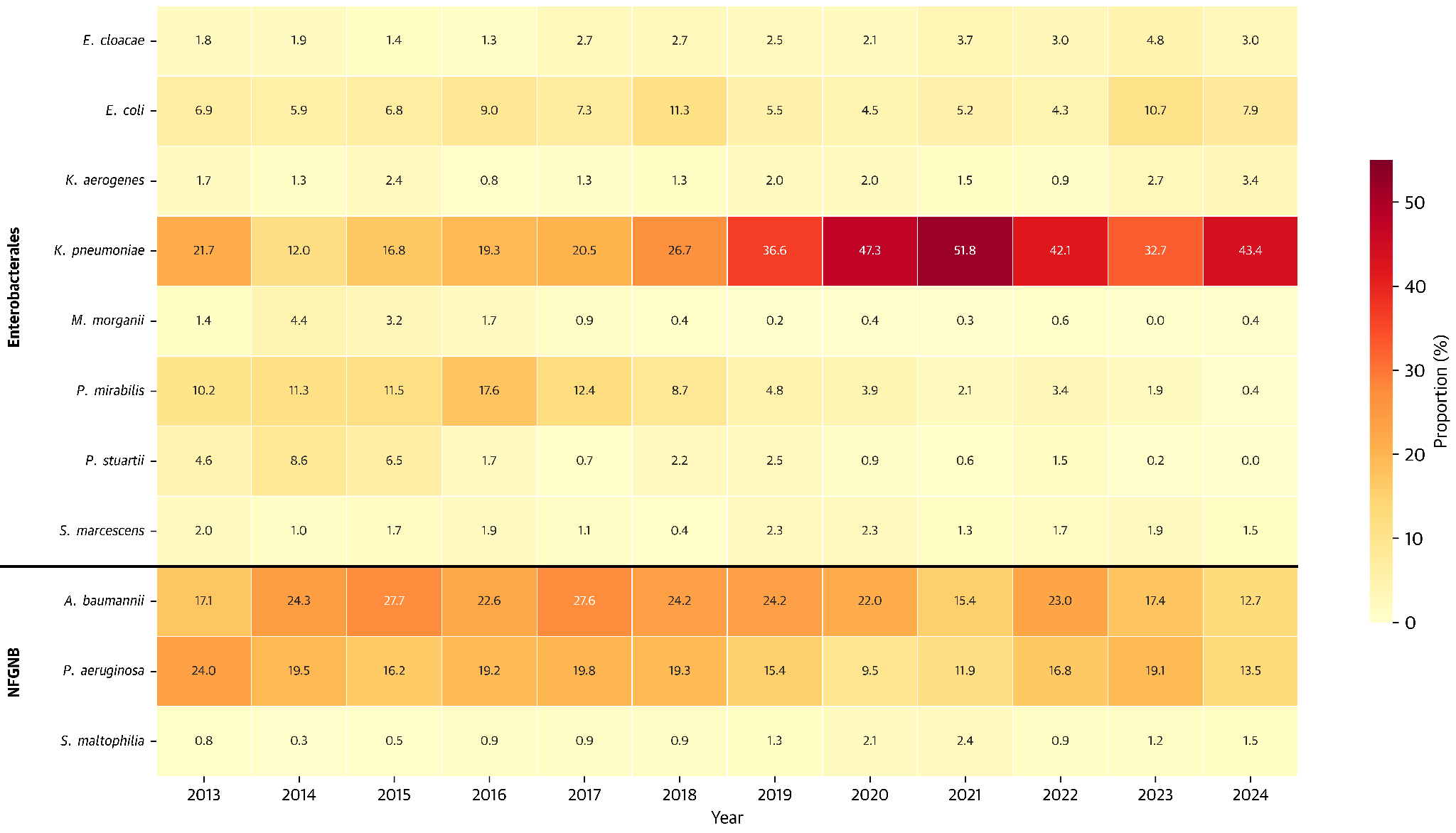


**eFigure 2.** Species-Specific Annual Proportions Among Respiratory Gram-Negative Bacilli. Heatmap displaying the annual proportion (%) of each species relative to all respiratory gram-negative bacilli isolates (N = 6,999). Abbreviations: GNB, gram-negative bacilli; NFGNB, non-fermenting gram-negative bacilli. Species: *A. baumannii*, *Acinetobacter baumannii*; *E. cloacae*, *Enterobacter cloacae*; *E. coli*, *Escherichia coli*; *K. aerogenes*, *Klebsiella aerogenes*; *K. pneumoniae*, *Klebsiella pneumoniae*; *M. morganii*, *Morganella morganii*; *P. aeruginosa*, *Pseudomonas aeruginosa*; *P. mirabilis*, *Proteus mirabilis*; *P. stuartii*, *Providencia stuartii*; *S. marcescens*, *Serratia marcescens*.


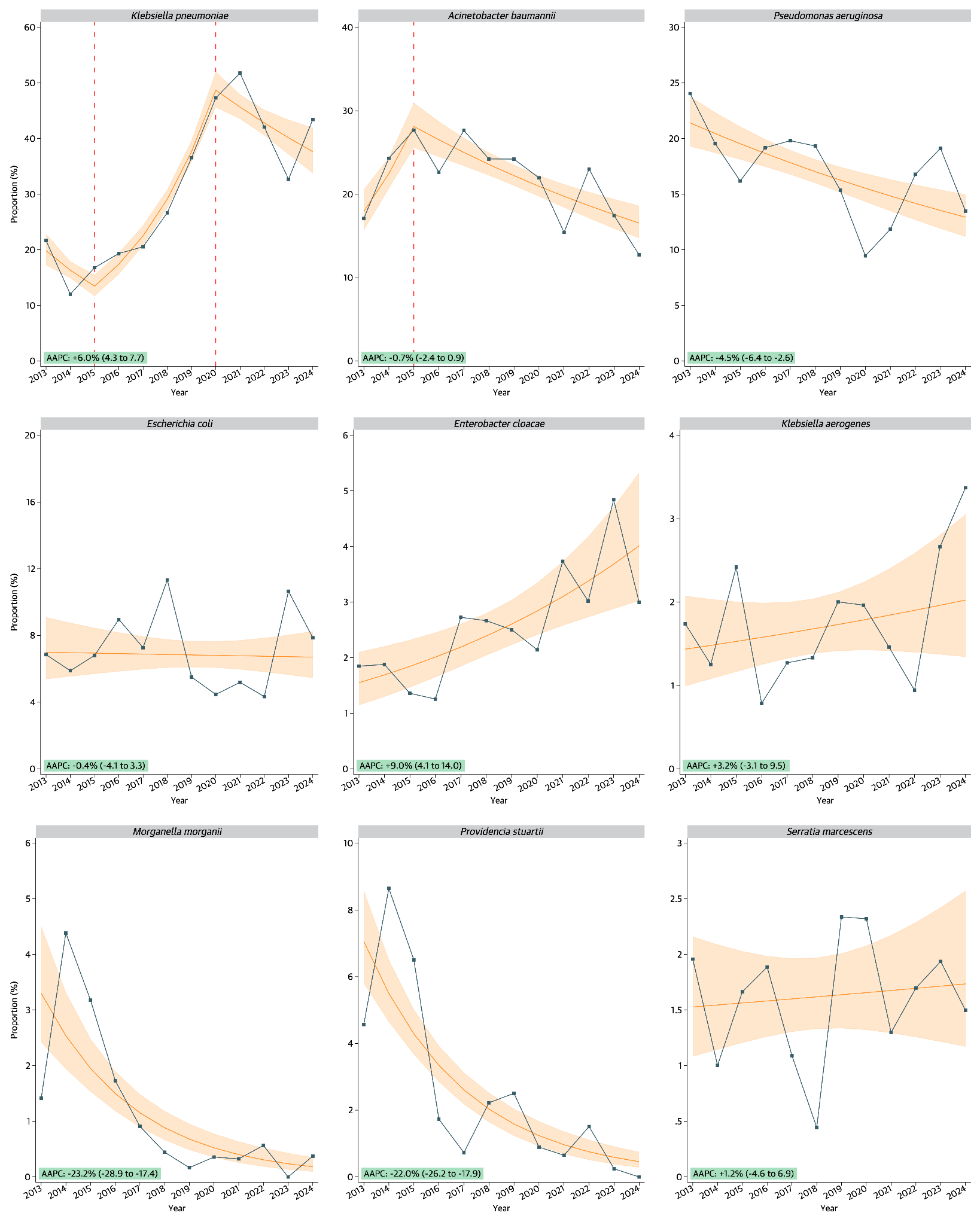


**eFigure 3.** Temporal Trends of Species-Level Proportions Among Respiratory Gram-Negative Bacilli. Observed proportions (connected markers) with segmented binomial regression trend lines and 95% confidence bands. Average annual percentage change (AAPC) with 95% confidence intervals is annotated in each panel. Vertical dashed lines indicate data-driven breakpoints.


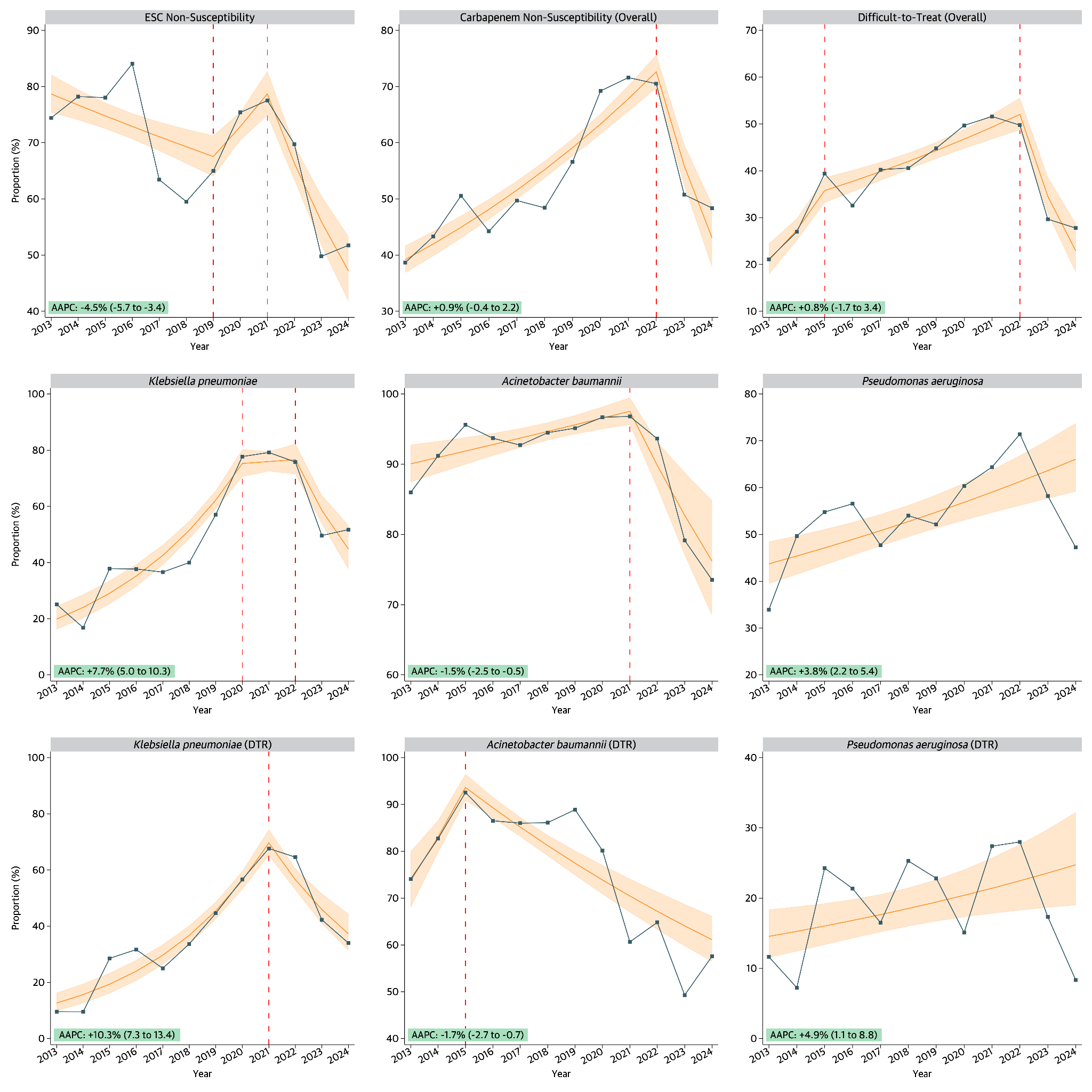


**eFigure 4.** Temporal Trends of Resistance Phenotype Proportions Among Respiratory Gram-Negative Bacilli. Observed proportions (connected markers) with segmented binomial regression trend lines and 95% confidence bands. Average annual percentage change (AAPC) with 95% confidence intervals is annotated in each panel. Vertical dashed lines indicate data-driven breakpoints. Abbreviations: AAPC, Average annual percentage change; CRAB, Carbapenem-resistant *Acinetobacter baumannii*; CRE, Carbapenem-resistant Enterobacterales; CRPA, Carbapenem-Resistant *Pseudomonas aeruginosa*; DTR, Difficult-to-treat.

**eTable 8.** Linearity Testing for Continuous Variables in 30-Day Mortality Models Among Respiratory Gram-Negative Bacilli, 2013–2024 ᵃ

| **Variable** | **Linear AIC** | **Alternative 1 AIC ᵇ** | **Alternative 2 AIC ᵇ** | **ΔAIC** | **P (non-linearity)** | **Chosen Form ᶜ** |
| --- | --- | --- | --- | --- | --- | --- |
| Age (per SD) | 3,901.3 | 3,897.6 | 3,898.8 | 3.7 | 0.002 | RCS(3) |
| Elixhauser comorbidity index (van Walraven) ᵈ | 3,901.2 | 3,900.1 | 3,902.0 | 1.1 | — | Linear |
| Year (calendar) | 3,901.3 | 3,896.6 | 3,898.8 | 4.7 | <0.001 | RCS(3) |
| Abbreviations: AIC, Akaike Information Criterion; ΔAIC, change in Akaike Information Criterion; RCS, restricted cubic spline; SD, standard deviation. | | | | | | |
| a—All models adjusted for: organism-resistance composite, age, sex, intensive care unit (ICU) at culture, concordant bacteremia, healthcare-associated infection, COVID-19 era, Elixhauser Comorbidity Index (ECI; van Walraven weighting), and calendar year.  b—For age and year: Alternative 1 = restricted cubic spline (RCS; 3 knots), Alternative 2 = RCS (4 knots). For Elixhauser Comorbidity Index (ECI): Alternative 1 = fractional polynomial (FP) degree 1, Alternative 2 = FP degree 2.  c—Selection rule: non-linear form preferred if change in Akaike Information Criterion (ΔAIC) > 2 (linear minus best alternative) or Wald P < 0.05 for non-linearity; otherwise linear retained (parsimony).  d—Elixhauser Comorbidity Index (ECI) tested via fractional polynomial (FP) instead of restricted cubic spline (RCS) due to zero-inflated distribution; FP handles concentrated data more robustly. Raw ECI scale used for FP testing; Winsorized standardized (per SD) form used when linear term selected. | | | | | | |


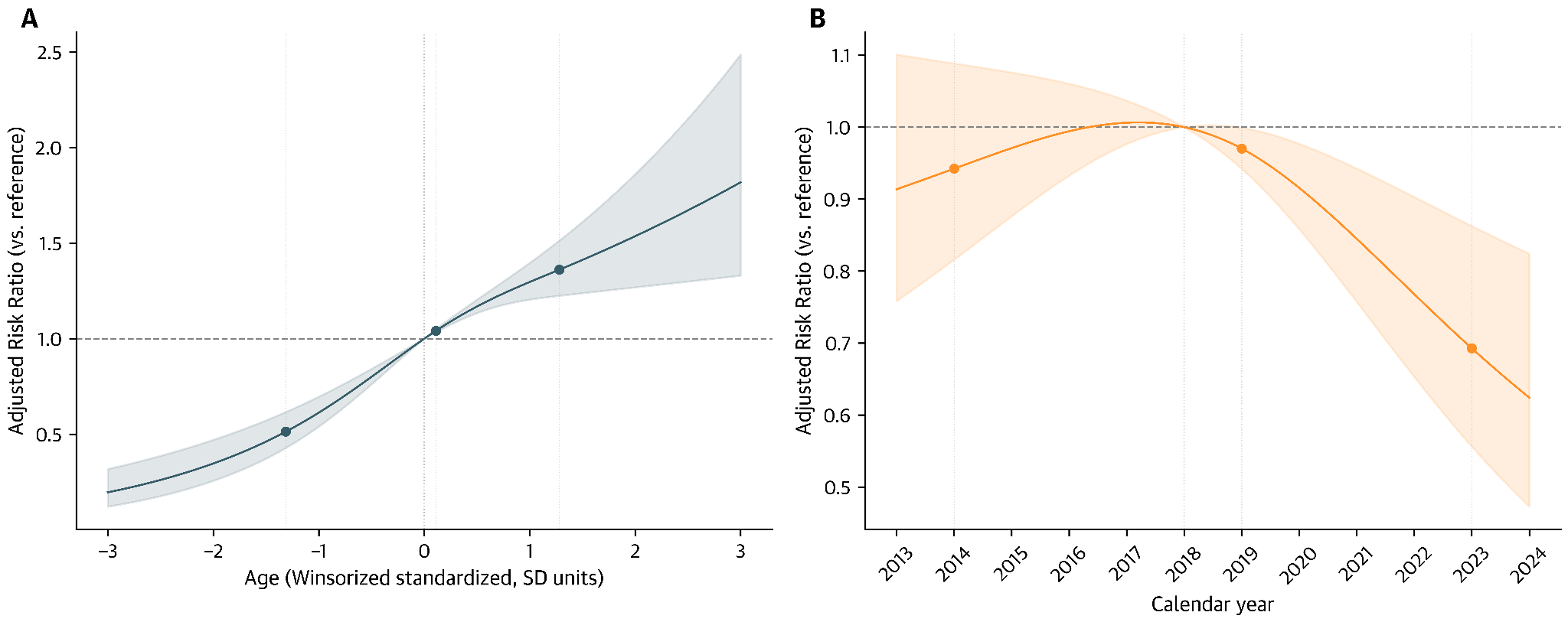


**eFigure 5.** Restricted Cubic Spline Dose-Response Curves for Continuous Covariables and 30-Day Mortality Among Respiratory Gram-Negative Bacilli. (A) Age (winsorized standardized, SD units) with reference at 0 (population mean). (B) Calendar year with reference to 2018. Curves are derived from the primary composite model (modified Poisson regression with robust standard errors clustered by patient) using 3 knot restricted cubic splines. Solid lines represent adjusted risk ratios; shaded bands represent 95% confidence intervals estimated from the variance covariance matrix. Dots indicate knot positions. Vertical dashed lines mark the reference value. The dashed horizontal line indicates the null value (adjusted risk ratio = 1.0). Abbreviations: SD, standard deviation.

**eTable 9.** Multivariable Predictors of 30-Day Mortality Among Respiratory Gram-Negative Bacilli, 2013–2024 ᵃ

| **Variable** | **N** | **Died n (%)** | **Crude RR (95% CI)** | **aRR (95% CI)** | **P value** |
| --- | --- | --- | --- | --- | --- |
| Organism-Resistance Composite | | | | | |
| Non-difficult-to-treat | | | | | |
| Enterobacterales | | | | | |
| Carbapenem-susceptible (ref.) | 1342 | 271 (20.2) | 1.00 | 1.00 | — |
| Carbapenem-resistant | 240 | 58 (24.2) | 1.20 (0.93 to 1.54) | 1.00 (0.81 to 1.24) | 0.972 |
| Non-fermenting GNB | | | | | |
| Carbapenem-susceptible | 381 | 62 (16.3) | 0.81 (0.63 to 1.04) | 0.84 (0.67 to 1.06) | 0.147 |
| Carbapenem-resistant | 234 | 66 (28.2) | 1.40 (1.11 to 1.76) | 1.07 (0.87 to 1.33) | 0.511 |
| Difficult-to-treat | | | | | |
| Enterobacterales | 562 | 253 (45.0) | 2.23 (1.94 to 2.57) | 1.34 (1.16 to 1.55) | <0.001 |
| Non-fermenting GNB | 856 | 305 (35.6) | 1.76 (1.53 to 2.03) | 1.28 (1.12 to 1.46) | <0.001 |
| Demographics | | | | | |
| Age ᵇ | 3615 | — | 1.57 (1.49 to 1.66) | Non-linear ᵇ | <0.001 |
| Women | 1073 | 360 (33.6) | 1.30 (1.17 to 1.45) | 1.28 (1.16 to 1.41) | <0.001 |
| Clinical Setting | | | | | |
| Intensive care unit at culture | 1738 | 851 (49.0) | 5.60 (4.80 to 6.54) | 4.58 (3.92 to 5.36) | <0.001 |
| Concordant bacteremia | 296 | 164 (55.4) | 2.16 (1.92 to 2.43) | 1.28 (1.14 to 1.43) | <0.001 |
| Healthcare-associated | 2666 | 822 (30.8) | 1.52 (1.32 to 1.74) | 1.07 (0.94 to 1.21) | 0.328 |
| Temporal | | | | | |
| COVID-19 era (2020–2024) | 1548 | 495 (32.0) | 1.27 (1.15 to 1.41) | 1.09 (0.92 to 1.29) | 0.307 |
| Year ᵇ | 3615 | — | 1.02 (1.00 to 1.04) | Non-linear ᵇ | 0.003 |
| Comorbidity | | | | | |
| Elixhauser Comorbidity Index (per SD) | 3615 | — | 1.06 (1.01 to 1.11) | 1.03 (0.99 to 1.07) | 0.164 |
| Abbreviations: aRR, adjusted risk ratio; CI, confidence interval; GNB, gram-negative bacilli; RCS, restricted cubic spline; RR, risk ratio; SD, standard deviation. | | | | | |
| a—30-day in-hospital mortality; restricted to first culture per admission (N = 3,615); modified Poisson regression with robust standard errors clustered by patient. Age and Elixhauser Comorbidity Index (ECI) entered as Winsorized standardized variables (per standard deviation); non-linear forms applied where indicated by linearity testing (eTable 8). Model Akaike Information Criterion (AIC) = 3,893.4.  b—Variables modeled with restricted cubic splines where non-linearity was detected; linearity testing results reported in eTable 8. Risk ratio (RR) not shown for non-linear terms; P value represents the Wald test for overall variable effect. | | | | | |


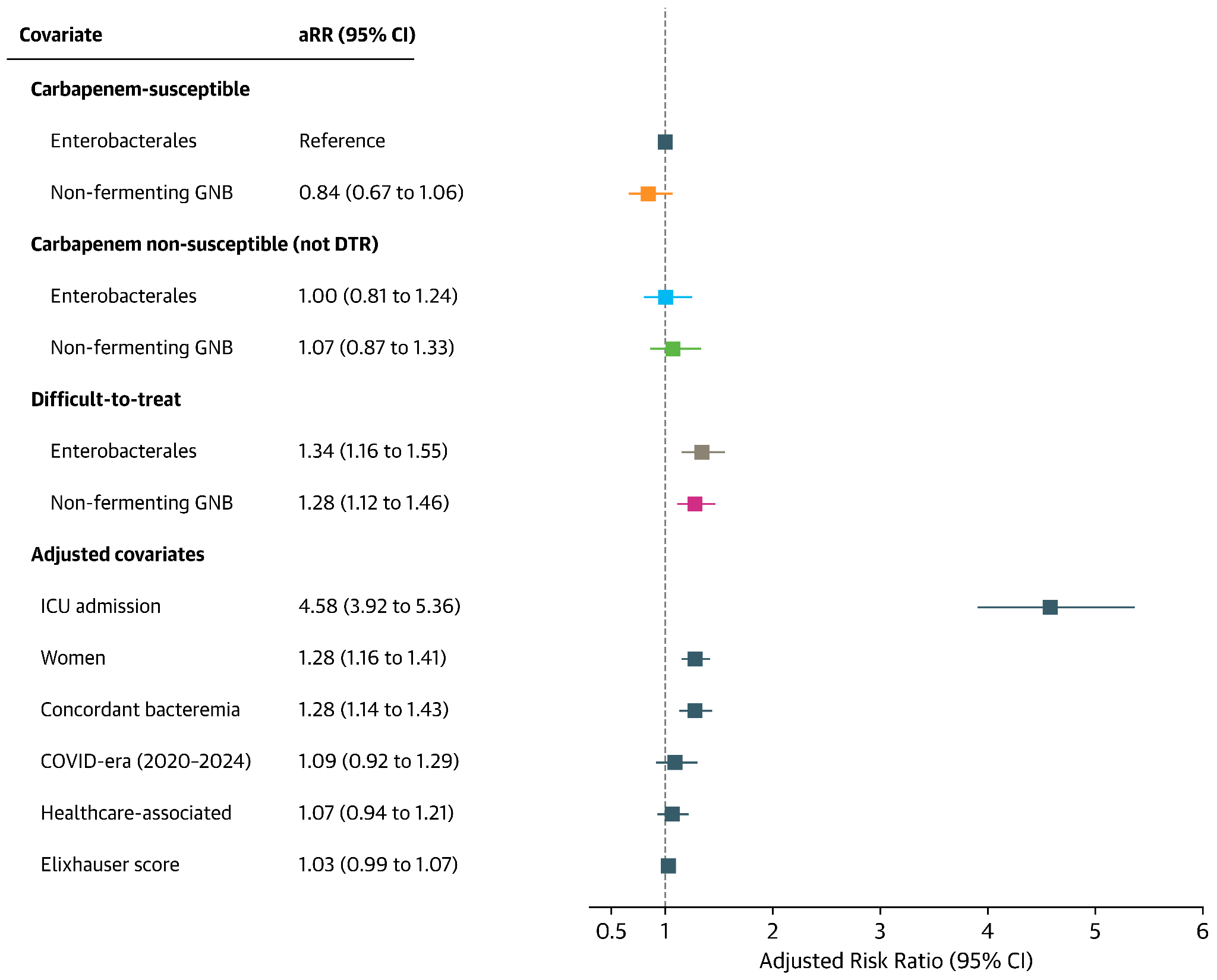


**eFigure 6.** Forest Plot of Adjusted Risk Ratios for 30-Day Mortality by Composite Resistance–Organism Classification Among Respiratory Gram-Negative Bacilli. Adjusted risk ratios with 95% confidence intervals (N = 3,615 first respiratory cultures per admission). The primary exposure is a six-level composite variable crossing organism group (Enterobacterales vs non-fermenting gram-negative bacilli) with resistance hierarchy (carbapenem-susceptible, carbapenem-resistant but not difficult-to-treat, and difficult-to-treat). Reference category: carbapenem-susceptible Enterobacterales. All models adjusted for age, sex, intensive care unit admission at culture, concordant bacteremia, healthcare-associated infection, COVID-19 era, year, and Elixhauser Comorbidity Index. Abbreviations: aRR, adjusted risk ratio; CI, confidence interval; DTR, difficult-to-treat resistance; GNB, gram-negative bacilli; ICU, intensive care unit.

**eTable 10.** Resistance Hierarchy and 30-Day Mortality Among Respiratory Gram-Negative Bacilli, 2013–2024 ᵃ

| **Variable** | **N** | **Died n (%)** | **Crude RR (95% CI)** | **aRR (95% CI)** | **P value** |
| --- | --- | --- | --- | --- | --- |
| Resistance Hierarchy ᵇ | | | | | |
| Non-resistant (ref.) | 1136 | 205 (18.0) | 1.00 | 1.00 | — |
| Fluoroquinolone-resistance | 297 | 71 (23.9) | 1.32 (1.04 to 1.68) | 1.20 (0.96 to 1.49) | 0.103 |
| Extended-spectrum cephalosporin-resistance ᶜ | 477 | 109 (22.9) | 1.27 (1.03 to 1.56) | 1.09 (0.89 to 1.34) | 0.391 |
| Carbapenem-resistance | 562 | 144 (25.6) | 1.42 (1.18 to 1.71) | 1.11 (0.94 to 1.31) | 0.236 |
| Difficult-to-treat | 1418 | 558 (39.4) | 2.18 (1.90 to 2.51) | 1.40 (1.22 to 1.60) | <0.001 |
| Organism Group | | | | | |
| Non-fermenting GNB (vs Enterobacterales) | 1652 | 483 (29.2) | 1.08 (0.98 to 1.20) | 0.98 (0.88 to 1.08) | 0.618 |
| Demographics | | | | | |
| Age ᵈ | 3890 | — | 1.56 (1.48 to 1.65) | Non-linear ᵈ | <0.001 |
| Women | 1175 | 387 (32.9) | 1.28 (1.15 to 1.42) | 1.26 (1.15 to 1.39) | <0.001 |
| Clinical Setting | | | | | |
| Intensive care unit at culture | 1860 | 908 (48.8) | 5.54 (4.78 to 6.41) | 4.60 (3.95 to 5.34) | <0.001 |
| Concordant bacteremia | 301 | 166 (55.1) | 2.15 (1.91 to 2.41) | 1.27 (1.14 to 1.42) | <0.001 |
| Healthcare-associated | 2890 | 884 (30.6) | 1.51 (1.32 to 1.73) | 1.06 (0.94 to 1.20) | 0.349 |
| Temporal | | | | | |
| COVID-19 era (2020–2024) | 1661 | 526 (31.7) | 1.26 (1.14 to 1.39) | 1.10 (0.93 to 1.29) | 0.253 |
| Year ᵈ | 3890 | — | 1.02 (1.00 to 1.03) | Non-linear ᵈ | <0.001 |
| Comorbidity | | | | | |
| Elixhauser Comorbidity Index (per SD) | 3890 | — | 1.06 (1.02 to 1.11) | 1.03 (0.99 to 1.07) | 0.163 |
| Abbreviations: aRR, adjusted risk ratio; CI, confidence interval; GNB, gram-negative bacilli; ICU, intensive care unit; RR, risk ratio; SD, standard deviation. | | | | | |
| a—30-day in-hospital mortality; restricted to first culture per admission (N = 3,890); modified Poisson regression with robust standard errors clustered by patient. Age and Elixhauser Comorbidity Index (ECI) entered as Winsorized standardized variables (per standard deviation); non-linear forms applied where indicated by linearity testing (eTable 8). Model Akaike Information Criterion (AIC) = 4,186.2.  b—Resistance classified using a 5-level mutually exclusive hierarchy: non-resistant, fluoroquinolone-resistant (FQR), extended-spectrum cephalosporin-resistant (ESC-R), carbapenem-resistant, and difficult-to-treat resistance (DTR) (Kadri et al., 2018).  c—ESC-R classification applies to Enterobacterales only; non-fermenting gram-negative bacilli (GNB) classified as non-resistant, FQR, carbapenem-resistant, or DTR.  d—Variables modeled with restricted cubic splines (3 knots) where non-linearity was detected; linearity testing results reported in eTable 8. Risk ratio (RR) not shown for non-linear terms; P value represents the Wald test for overall variable effect. | | | | | |

**eTable 11.** First-Line Active Agents and 30-Day Mortality Among Respiratory Gram-Negative Bacilli, 2013–2024 ᵃ ᵇ

| **Variable** | **N** | **Died n (%)** | **Crude RR (95% CI)** | **aRR (95% CI)** | **P value** |
| --- | --- | --- | --- | --- | --- |
| First-Line Active Categories ᶜ ᵈ | | | | | |
| Adequate (3–5 active; ref.) | 1293 | 225 (17.4) | 1.00 | 1.00 | — |
| Limited (1–2 active) | 683 | 187 (27.4) | 1.57 (1.33 to 1.87) | 1.22 (1.04 to 1.43) | 0.014 |
| Difficult-to-treat (0 active) | 1416 | 558 (39.4) | 2.26 (1.98 to 2.59) | 1.47 (1.28 to 1.67) | <0.001 |
| Organism Group | | | | | |
| Non-fermenting GNB (vs Enterobacterales) | 1455 | 431 (29.6) | 1.06 (0.96 to 1.19) | 0.95 (0.86 to 1.05) | 0.300 |
| Demographics | | | | | |
| Age ᵉ | 3392 | — | 1.58 (1.50 to 1.67) | Non-linear ᵉ | <0.001 |
| Women | 1004 | 342 (34.1) | 1.30 (1.16 to 1.45) | 1.26 (1.15 to 1.39) | <0.001 |
| Clinical Setting | | | | | |
| Intensive care unit at culture | 1661 | 821 (49.4) | 5.74 (4.89 to 6.75) | 4.68 (3.97 to 5.51) | <0.001 |
| Concordant bacteremia | 285 | 159 (55.8) | 2.14 (1.90 to 2.41) | 1.24 (1.11 to 1.39) | <0.001 |
| Healthcare-associated | 2522 | 793 (31.4) | 1.55 (1.34 to 1.79) | 1.06 (0.93 to 1.21) | 0.386 |
| Temporal | | | | | |
| COVID-19 era (2020–2024) | 1450 | 474 (32.7) | 1.28 (1.15 to 1.42) | 1.07 (0.91 to 1.27) | 0.420 |
| Year ᵉ | 3392 | — | 1.02 (1.01 to 1.04) | Non-linear ᵉ | 0.002 |
| Comorbidity | | | | | |
| Elixhauser Comorbidity Index (per SD) | 3392 | — | 1.06 (1.01 to 1.11) | 1.03 (0.98 to 1.07) | 0.245 |
| Abbreviations: aRR, adjusted risk ratio; CI, confidence interval; COVID, coronavirus disease 2019; GNB, gram-negative bacilli; RR, risk ratio; SD, standard deviation. | | | | | |
| a—30-day in-hospital mortality; restricted to first culture per admission (N = 3,392); modified Poisson regression with robust standard errors clustered by patient. Age and Elixhauser Comorbidity Index (ECI) entered as Winsorized standardized variables (per standard deviation); non-linear forms applied where indicated by linearity testing (eTable 8). Model Akaike Information Criterion (AIC) = 3,677.3.  b—N = 3,392 restricted to isolates with susceptibility data for ≥3 mandatory first-line categories (carbapenems, extended-spectrum cephalosporins, fluoroquinolones); piperacillin-tazobactam and the 5th agent counted when reported. 554 admissions excluded due to incomplete susceptibility data.  c—Five first-line antibiotic categories were evaluated for activity: (1) carbapenems (imipenem, meropenem, ertapenem), (2) extended-spectrum cephalosporins (ceftriaxone, cefotaxime, ceftazidime, cefepime), (3) fluoroquinolones (ciprofloxacin, levofloxacin), (4) piperacillin-tazobactam (when tested), and (5) aztreonam for non-*Acinetobacter* species or ampicillin-sulbactam for *Acinetobacter baumannii* (when tested). Analysis restricted to Enterobacterales, *Pseudomonas aeruginosa*, and *Acinetobacter baumannii* (reference group: ≥3 active categories; Kadri et al., 2018).  d—P-for-trend < 0.001 (ordinal contrast across active-category groups).  e—Variables modeled with restricted cubic splines (3 knots) where non-linearity was detected; linearity testing results reported in eTable 8. Risk ratio (RR) not shown for non-linear terms; P value represents the Wald test for overall variable effect. | | | | | |


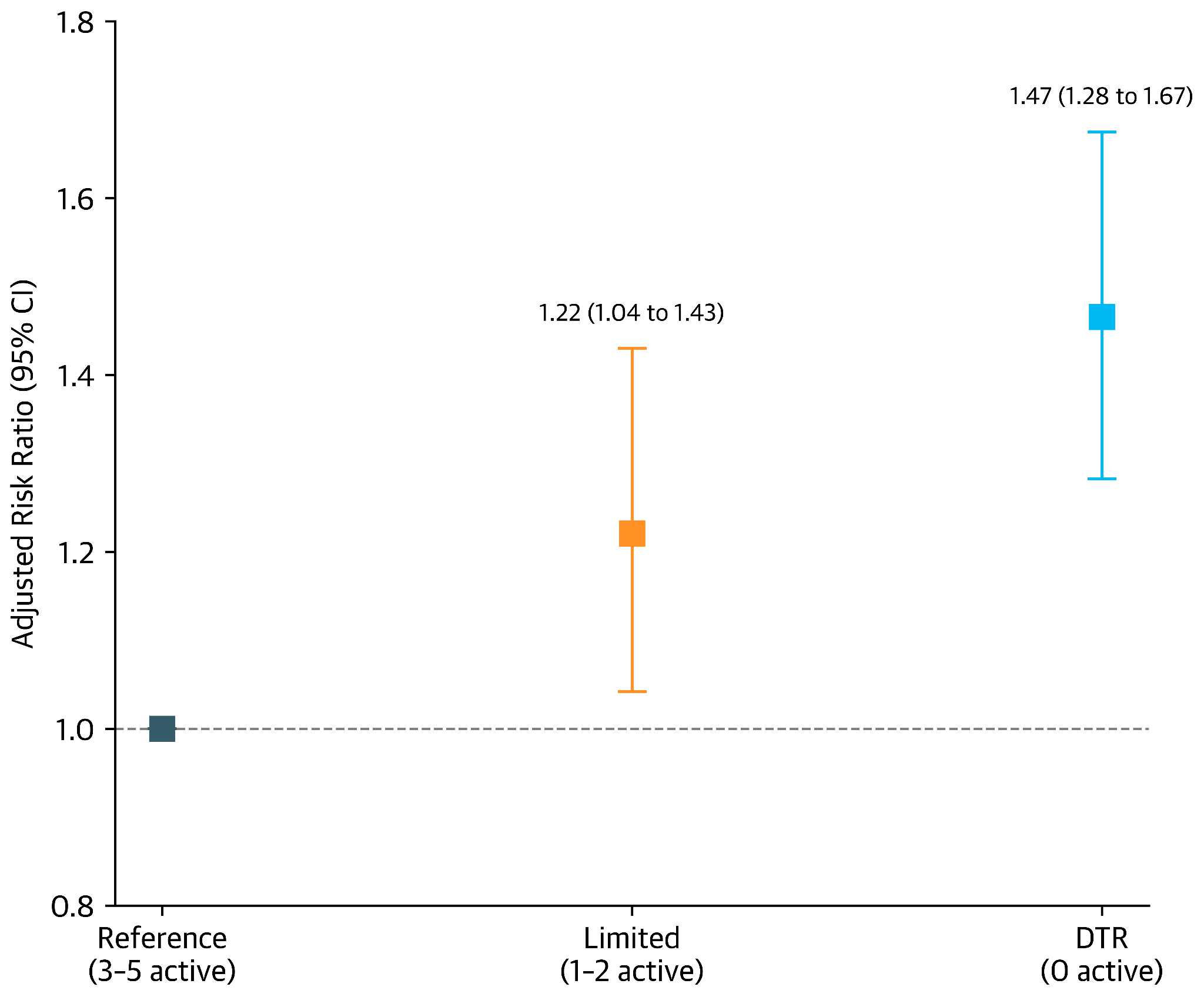


**eFigure 7.** Dose-Response Relationship Between First-Line Active Antimicrobial Categories and 30-Day Mortality Among Respiratory Gram-Negative Bacilli. Adjusted risk ratios with 95% confidence intervals for 30-day mortality across three levels of first-line active agent availability: adequate (3–5 active categories; reference), limited (1–2 active), and difficult-to-treat resistance (0 active). First-line categories counted are carbapenems, extended-spectrum cephalosporins, fluoroquinolones, piperacillin-tazobactam, and aztreonam (or ampicillin-sulbactam for *Acinetobacter baumannii*). Modified Poisson regression adjusted for organism group, age, sex, intensive care unit admission at culture, concordant bacteremia, healthcare-associated infection, COVID-19 era, Elixhauser Comorbidity Index, and year. Abbreviations: CI, confidence interval.

**eTable 12.** Sensitivity and Alternative Model Analyses for 30-Day Mortality Among Respiratory Gram-Negative Bacilli, 2013–2024 ᵃ

| **Part A:** Composite Exposure Across Sensitivity Specifications, aRR (95% CI) | | | | | | |
| --- | --- | --- | --- | --- | --- | --- |
| Variable | A: Primary | B: In-hospital | C: First admission | D: No ICU | E: No BSI | F: Last culture |
| Non-difficult-to-treat | | | | | | |
| Carbapenem-resistant Enterobacterales | 1.00 (0.81 to 1.24) | 0.93 (0.77 to 1.12) | 1.00 (0.81 to 1.24) | 1.11 (0.87 to 1.42) | 1.03 (0.83 to 1.27) | 0.94 (0.79 to 1.13) |
| Carbapenem-susceptible non-fermenting GNB | 0.84 (0.67 to 1.06) | 0.95 (0.80 to 1.13) | 0.85 (0.68 to 1.07) | 0.83 (0.65 to 1.06) | 0.84 (0.67 to 1.06) | 0.80 (0.66 to 0.98) |
| Carbapenem-resistant non-fermenting GNB | 1.07 (0.87 to 1.33) | 1.13 (0.95 to 1.34) | 1.10 (0.89 to 1.36) | 1.32 (1.06 to 1.65) | 1.08 (0.88 to 1.34) | 1.01 (0.85 to 1.20) |
| Difficult-to-treat | | | | | | |
| Enterobacterales | 1.34 (1.16 to 1.55) | 1.32 (1.17 to 1.48) | 1.32 (1.14 to 1.52) | 1.72 (1.48 to 2.00) | 1.42 (1.24 to 1.63) | 1.26 (1.12 to 1.43) |
| Non-fermenting GNB | 1.28 (1.12 to 1.46) | 1.25 (1.12 to 1.39) | 1.29 (1.13 to 1.47) | 1.69 (1.47 to 1.94) | 1.29 (1.13 to 1.47) | 1.33 (1.19 to 1.48) |
| Model G (resistance hierarchy × organism group interaction): Wald test P = 0.447. Interaction terms not tabulated; organism-stratified estimates in the exported coefficient dataset. | | | | | | |
| **Part B:** Staged Regression Decomposition (Composite Model) ᵇ | | | | | | |
| Variable | S1: Base | S2: +ICU | S3: +BSI (full) |  |  |  |
| Difficult-to-treat | | | |  |  |  |
| Enterobacterales | 1.88 (1.62 to 2.17) | 1.42 (1.24 to 1.63) | 1.34 (1.16 to 1.55) |  |  |  |
| Non-fermenting GNB | 1.73 (1.51 to 1.99) | 1.29 (1.13 to 1.47) | 1.28 (1.12 to 1.46) |  |  |  |
| Abbreviations: aRR, adjusted risk ratio; BSI, bloodstream infection; CI, confidence interval; GNB, gram-negative bacilli; ICU, intensive care unit. | | | | | | |
| a—All models: modified Poisson regression with exponentiated form (adjusted risk ratio [aRR]). Reference group: carbapenem-susceptible Enterobacterales. Clustered by patient (except Model C: robust standard errors, 1 observation/patient).  b—S1 = age, sex, healthcare-associated infection, COVID-19 era, calendar year, Elixhauser Comorbidity Index (ECI). S2 = S1 + intensive care unit (ICU) at culture. S3 = S2 + concordant bacteremia (full model). | | | | | | |


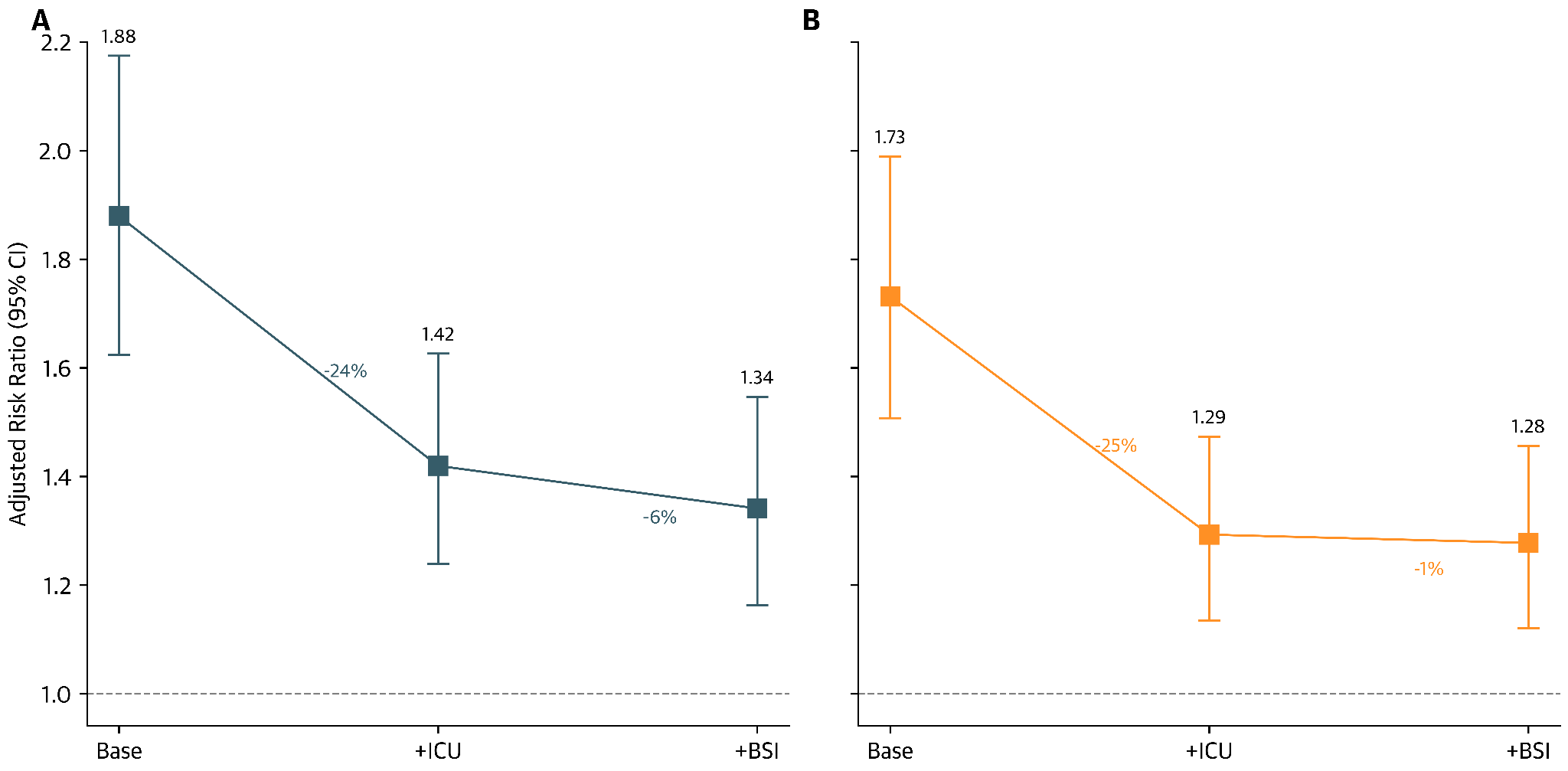


**eFigure 8.** Staged Regression Decomposition of Difficult-to-Treat Resistance and 30-Day Mortality Among Respiratory Gram-Negative Bacilli. Connected dot plots showing the attenuation of adjusted risk ratios across three nested models for the two difficult-to-treat resistance (DTR) categories: (A) DTR Enterobacterales and (B) DTR non-fermenting gram-negative bacilli. All models use modified Poisson regression with robust standard errors clustered by patient. Stage 1 (Base) adjusts for age, sex, healthcare-associated infection, COVID-19 era, year, and Elixhauser Comorbidity Index. Stage 2 (+ICU) adds intensive care unit admission at culture. Stage 3 (+BSI) adds concordant bacteremia (full model). Percentage annotations between stages indicate the proportional change in the risk ratio point estimate. Reference category: carbapenem-susceptible Enterobacterales. Abbreviations: BSI, bloodstream infection; CI, confidence interval.


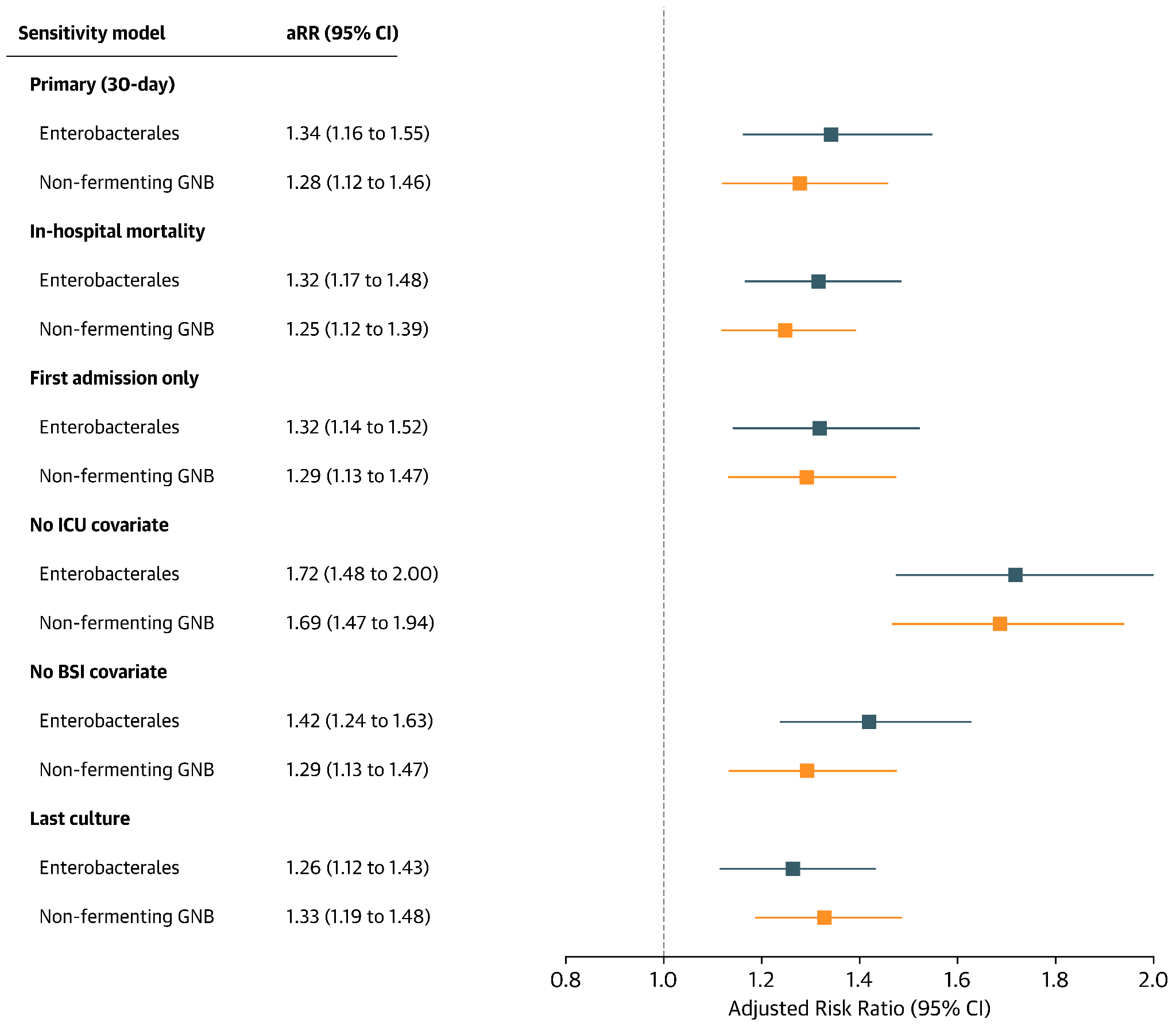


**eFigure 9.** Sensitivity Analysis Comparison of Difficult-to-Treat Resistance and 30-Day Mortality Across Model Specifications Among Respiratory Gram-Negative Bacilli. Forest plot comparing adjusted risk ratios for difficult-to-treat Enterobacterales and difficult-to-treat non-fermenting gram-negative bacilli across six model specifications: primary model (30-day mortality), in-hospital mortality as outcome, first admission only (one observation per patient, robust standard errors), intensive care unit covariable removed, concordant bacteremia covariable removed, and last culture per admission instead of first. All models use modified Poisson regression with the six-level composite exposure variable. Reference category: carbapenem-susceptible Enterobacterales. Abbreviations: aRR, adjusted risk ratio; BSI, bloodstream infection; CI, confidence interval; GNB, gram-negative bacilli; ICU, intensive care unit.

**eTable 13.** Subgroup Trend Analyses (AAPC) by Sex and ICU Status Among Respiratory Gram-Negative Bacilli, 2013–2024

| **Category** | **Sex (AAPC% [95% CI])** | | |  | **Intensive care unit (AAPC% [95% CI])** | | |
| --- | --- | --- | --- | --- | --- | --- | --- |
|  | **Men** | **Women** | **ΔAAPC (Men – Women; % [95% CI])** |  | **ICU** | **Non-ICU** | **ΔAAPC**  **(ICU – Non-ICU; % [95% CI])** |
| Organism proportion | | | | | | | |
| Enterobacterales | 1.8 (0.9 to 2.7) | 2.3 (0.9 to 3.8) | -0.5 (-2.2 to 1.2) |  | 1.4 (0.3 to 2.5) | 2.7 (1.7 to 3.7) | -1.3 (-2.8 to 0.2) |
| Non-fermenting GNB | -2.4 (-3.6 to -1.2) | -2.7 (-4.3 to -1.1) | 0.2 (-1.7 to 2.2) |  | -1.7 (-3.0 to -0.4) | -3.8 (-5.1 to -2.4) | 2.1 (0.2 to 4.0) |
| Resistance phenotypes | | | | | | | |
| Fluoroquinolone resistance | -4.3 (-5.6 to -3.0) | -2.7 (-4.0 to -1.5) | -1.6 (-3.4 to 0.2) |  | -5.4 (-7.0 to -3.8) | -2.6 (-3.9 to -1.4) | -2.8 (-4.8 to -0.7) |
| Extended-spectrum cephalosporin resistance | -4.6 (-6.0 to -3.2) | -2.1 (-3.2 to -1.0) | -2.5 (-4.3 to -0.7) |  | -3.3 (-4.9 to -1.6) | -4.0 (-5.3 to -2.6) | 0.7 (-1.4 to 2.8) |
| Carbapenem-resistant Enterobacterales | 9.5 (7.3 to 11.7) | 3.2 (0.2 to 6.2) | 6.3 (2.6 to 10.1) |  | 11.5 (9.3 to 13.7) | 1.7 (-1.5 to 4.9) | 9.8 (5.9 to 13.7) |
| Carbapenem resistance (overall) | 0.8 (-0.8 to 2.3) | 2.1 (0.3 to 4.0) | -1.4 (-3.8 to 1.1) |  | 4.0 (2.8 to 5.3) | -2.7 (-5.0 to -0.5) | 6.8 (4.2 to 9.4) |
| Difficult-to-treat resistance | 1.2 (-0.6 to 3.1) | -0.6 (-5.8 to 4.7) | 1.8 (-3.8 to 7.4) |  | 3.3 (-0.0 to 6.7) | -3.7 (-6.6 to -0.8) | 7.0 (2.6 to 11.4) |
| Top species | | | | | | | |
| *Acinetobacter baumannii* | -2.1 (-3.8 to -0.4) | 0.3 (-2.7 to 3.2) | -2.4 (-5.8 to 1.0) |  | 1.5 (-0.9 to 3.8) | -4.5 (-6.9 to -2.2) | 6.0 (2.7 to 9.3) |
| *Escherichia coli* | -2.0 (-6.6 to 2.7) | 2.8 (-2.7 to 8.3) | -4.8 (-12.0 to 2.5) |  | -7.5 (-13.2 to -1.7) | 6.5 (2.8 to 10.1) | -13.9 (-20.7 to -7.1) |
| *Klebsiella pneumoniae* | 5.3 (3.3 to 7.2) | 8.5 (5.3 to 11.7) | -3.2 (-7.0 to 0.5) |  | 7.7 (5.2 to 10.3) | 4.3 (2.2 to 6.5) | 3.4 (0.1 to 6.8) |
| *Proteus mirabilis* | -17.2 (-23.3 to -11.2) | -20.6 (-26.6 to -14.6) | 3.4 (-5.1 to 11.9) |  | -15.5 (-20.6 to -10.4) | -18.4 (-23.5 to -13.3) | 2.9 (-4.3 to 10.1) |
| *Pseudomonas aeruginosa* | -3.8 (-6.1 to -1.6) | -5.6 (-8.9 to -2.4) | 1.8 (-2.2 to 5.8) |  | -4.6 (-7.3 to -2.0) | -4.3 (-6.9 to -1.6) | -0.4 (-4.1 to 3.4) |
| Abbreviations: AAPC, average annual percentage change; CI, confidence interval; GNB, gram-negative bacilli; ICU, intensive care unit; ΔAAPC, difference in AAPC between subgroups. | | | | | | | |

**eTable 14.** Trend Analysis Feasibility Assessment Among Respiratory Gram-Negative Bacilli, 2013–2024

| **Outcome** | **Total Nᵃ** | **Events (%)ᵇ** |  | **Sex** | | **Intensive care unit** | |  | **Trend analysisᶜ** | | |
| --- | --- | --- | --- | --- | --- | --- | --- | --- | --- | --- | --- |
|  |  |  |  | **Men** | **Women** | **ICU** | **Non-ICU** |  | **Overallᵈ** | **Sexᵉ** | **Settingᵉ** |
| Organism proportions | | | | | | | | | | | |
| Enterobacterales | 6,999 | 4002 (57.2%) |  | 2,857 | 1,145 | 2,105 | 1,897 |  | Included | Included | Included |
| Non-fermenting GNB | 6,999 | 2997 (42.8%) |  | 2,074 | 923 | 1,673 | 1,324 |  | Included | Included | Included |
| Species proportions | | | | | | | | | | | |
| Enterobacterales | | | | | | | | | | | |
| *Enterobacter cloacae* | 6,999 | 170 (2.4%) |  | 115 | 55 | 67 | 103 |  | Included | Included | Included |
| *Escherichia coli* | 6,999 | 481 (6.9%) |  | 332 | 149 | 215 | 266 |  | Included | Included | Included |
| *Klebsiella aerogenes* | 6,999 | 117 (1.7%) |  | 91 | 26 | 53 | 64 |  | Included | Excluded (Women < 50) | Included |
| *Klebsiella pneumoniae* | 6,999 | 2039 (29.1%) |  | 1,476 | 563 | 1,110 | 929 |  | Included | Included | Included |
| *Morganella morganii* | 6,999 | 96 (1.4%) |  | 74 | 22 | 67 | 29 |  | Included | Excluded (Women < 50) | Excluded (Non-ICU < 50) |
| *Proteus mirabilis* | 6,999 | 570 (8.1%) |  | 416 | 154 | 293 | 277 |  | Included | Included | Included |
| *Providencia stuartii* | 6,999 | 212 (3.0%) |  | 138 | 74 | 122 | 90 |  | Included | Included | Included |
| *Serratia marcescens* | 6,999 | 113 (1.6%) |  | 83 | 30 | 71 | 42 |  | Included | Excluded (Women < 50) | Excluded (Non-ICU < 50) |
| Non-fermenting GNB | | | | | | | | | | | |
| *Acinetobacter baumannii* | 6,999 | 1530 (21.9%) |  | 1,091 | 439 | 908 | 622 |  | Included | Included | Included |
| *Pseudomonas aeruginosa* | 6,999 | 1224 (17.5%) |  | 822 | 402 | 642 | 582 |  | Included | Included | Included |
| *Stenotrophomonas maltophilia* | 6,999 | 76 (1.1%) |  | 48 | 28 | 39 | 37 |  | Included | Excluded (Men < 50, Women < 50) | Excluded (ICU < 50, Non-ICU < 50) |
| Non-susceptible phenotypes | | | | | | | | | | | |
| Fluoroquinolone non-susceptible | 6,688 | 4572 (68.4%) |  | 3,190 | 1,382 | 2,637 | 1,935 |  | Included | Included | Included |
| Extended-spectrum cephalosporin resistance | 3,570 | 2558 (71.7%) |  | 1,804 | 754 | 1,474 | 1,084 |  | Included | Included | Included |
| Carbapenem-resistant Enterobacterales | 3,784 | 1421 (37.6%) |  | 995 | 426 | 869 | 552 |  | Included | Included | Included |
| Carbapenem resistance (overall) | 6,492 | 3428 (52.8%) |  | 2,412 | 1,016 | 2,070 | 1,358 |  | Included | Included | Included |
| Difficult-to-treat resistance (Enterobacterales) | 3,348 | 904 (27.0%) |  | 636 | 268 | 583 | 321 |  | Included | Included | Included |
| Difficult-to-treat resistance (overall) | 6,003 | 2259 (37.6%) |  | 1,596 | 663 | 1,390 | 869 |  | Included | Included | Included |
| Species-specific resistance | | | | | | | | | | | |
| Carbapenem-resistant isolates | | | | | | | | | | | |
| *Acinetobacter baumannii* | 1,503 | 1386 (92.2%) |  | 992 | 394 | 836 | 550 |  | Included | Included | Included |
| *Klebsiella pneumoniae* | 1,989 | 1091 (54.9%) |  | 758 | 333 | 693 | 398 |  | Included | Included | Included |
| *Pseudomonas aeruginosa* | 1,205 | 621 (51.5%) |  | 425 | 196 | 365 | 256 |  | Included | Included | Included |
| Difficult-to-treat resistance | | | | | | | | | | | |
| *Acinetobacter baumannii* | 1,438 | 1143 (79.5%) |  | 822 | 321 | 681 | 462 |  | Included | Included | Included |
| *Klebsiella pneumoniae* | 1,807 | 776 (42.9%) |  | 531 | 245 | 505 | 271 |  | Included | Included | Included |
| *Pseudomonas aeruginosa* | 1,147 | 208 (18.1%) |  | 135 | 73 | 125 | 83 |  | Included | Included | Included |
| Abbreviations: GNB, gram-negative bacilli; ICU, intensive care unit. | | | | | | | | | | | |
| a—Total N = evaluable isolates (nonmissing outcome).  b—Events = isolates positive for the outcome.  c—Gating criteria applied to all trend analyses (overall, sex, and setting): N ≥ 100 evaluable isolates, prevalence 0.5–99.5%.  d—Included if events ≥ 50.  e—Included if events ≥ 50 in both strata of each comparison (men and women; intensive care unit [ICU] and non-ICU). | | | | | | | | | | | |

**eTable 15.** Variable Completeness by Organism Group Among Respiratory Gram-Negative Bacilli, 2013–2024

| **Variable** | **Overall (N = 6,999) ᵃ** | **Organism Group** | |
| --- | --- | --- | --- |
|  |  | **Enterobacterales (n = 4,002) ᵃ** | **Non-fermenting GNB (n = 2,997) ᵃ** |
| Demographics | | | |
| Age, years | 6,999/6,999 (100.0%) | 4,002/4,002 (100.0%) | 2,997/2,997 (100.0%) |
| Sex | 6,999/6,999 (100.0%) | 4,002/4,002 (100.0%) | 2,997/2,997 (100.0%) |
| Sample received date | 6,999/6,999 (100.0%) | 4,002/4,002 (100.0%) | 2,997/2,997 (100.0%) |
| Species | 6,999/6,999 (100.0%) | 4,002/4,002 (100.0%) | 2,997/2,997 (100.0%) |
| Clinical setting | | | |
| Ward at culture collection | 6,999/6,999 (100.0%) | 4,002/4,002 (100.0%) | 2,997/2,997 (100.0%) |
| ICU at culture collection | 6,999/6,999 (100.0%) | 4,002/4,002 (100.0%) | 2,997/2,997 (100.0%) |
| Admission and outcomes | | | |
| Admission date | 6,999/6,999 (100.0%) | 4,002/4,002 (100.0%) | 2,997/2,997 (100.0%) |
| Discharge date | 6,999/6,999 (100.0%) | 4,002/4,002 (100.0%) | 2,997/2,997 (100.0%) |
| Length of stay, days | 6,999/6,999 (100.0%) | 4,002/4,002 (100.0%) | 2,997/2,997 (100.0%) |
| In-hospital mortality | 6,999/6,999 (100.0%) | 4,002/4,002 (100.0%) | 2,997/2,997 (100.0%) |
| 30-day in-hospital mortality | 6,999/6,999 (100.0%) | 4,002/4,002 (100.0%) | 2,997/2,997 (100.0%) |
| Concordant bacteremia | 6,999/6,999 (100.0%) | 4,002/4,002 (100.0%) | 2,997/2,997 (100.0%) |
| Comorbidity indices | | | |
| Elixhauser Comorbidity Index (unweighted) | 6,999/6,999 (100.0%) | 4,002/4,002 (100.0%) | 2,997/2,997 (100.0%) |
| van Walraven weighted Elixhauser score | 6,999/6,999 (100.0%) | 4,002/4,002 (100.0%) | 2,997/2,997 (100.0%) |
| Elixhauser comorbidity categories | | | |
| Autoimmune/connective tissue diseases | 6,999/6,999 (100.0%) | 4,002/4,002 (100.0%) | 2,997/2,997 (100.0%) |
| Cardiac arrhythmias | 6,999/6,999 (100.0%) | 4,002/4,002 (100.0%) | 2,997/2,997 (100.0%) |
| Chronic liver disease | 6,999/6,999 (100.0%) | 4,002/4,002 (100.0%) | 2,997/2,997 (100.0%) |
| Chronic pulmonary disease | 6,999/6,999 (100.0%) | 4,002/4,002 (100.0%) | 2,997/2,997 (100.0%) |
| Coagulopathy | 6,999/6,999 (100.0%) | 4,002/4,002 (100.0%) | 2,997/2,997 (100.0%) |
| Congestive heart failure | 6,999/6,999 (100.0%) | 4,002/4,002 (100.0%) | 2,997/2,997 (100.0%) |
| Deficiency anemia | 6,999/6,999 (100.0%) | 4,002/4,002 (100.0%) | 2,997/2,997 (100.0%) |
| Depression | 6,999/6,999 (100.0%) | 4,002/4,002 (100.0%) | 2,997/2,997 (100.0%) |
| Diabetes mellitus (complicated) | 6,999/6,999 (100.0%) | 4,002/4,002 (100.0%) | 2,997/2,997 (100.0%) |
| Diabetes mellitus (uncomplicated) | 6,999/6,999 (100.0%) | 4,002/4,002 (100.0%) | 2,997/2,997 (100.0%) |
| Fluid/electrolyte disorders | 6,999/6,999 (100.0%) | 4,002/4,002 (100.0%) | 2,997/2,997 (100.0%) |
| HIV/AIDS | 6,999/6,999 (100.0%) | 4,002/4,002 (100.0%) | 2,997/2,997 (100.0%) |
| Hypertension (complicated) | 6,999/6,999 (100.0%) | 4,002/4,002 (100.0%) | 2,997/2,997 (100.0%) |
| Hypertension (uncomplicated) | 6,999/6,999 (100.0%) | 4,002/4,002 (100.0%) | 2,997/2,997 (100.0%) |
| Hypothyroidism | 6,999/6,999 (100.0%) | 4,002/4,002 (100.0%) | 2,997/2,997 (100.0%) |
| Lymphoma/leukemia | 6,999/6,999 (100.0%) | 4,002/4,002 (100.0%) | 2,997/2,997 (100.0%) |
| Metastatic cancer | 6,999/6,999 (100.0%) | 4,002/4,002 (100.0%) | 2,997/2,997 (100.0%) |
| Obesity | 6,999/6,999 (100.0%) | 4,002/4,002 (100.0%) | 2,997/2,997 (100.0%) |
| Other neurological disorders | 6,999/6,999 (100.0%) | 4,002/4,002 (100.0%) | 2,997/2,997 (100.0%) |
| Paralysis | 6,999/6,999 (100.0%) | 4,002/4,002 (100.0%) | 2,997/2,997 (100.0%) |
| Peptic ulcer disease | 6,999/6,999 (100.0%) | 4,002/4,002 (100.0%) | 2,997/2,997 (100.0%) |
| Peripheral vascular disease | 6,999/6,999 (100.0%) | 4,002/4,002 (100.0%) | 2,997/2,997 (100.0%) |
| Psychoses | 6,999/6,999 (100.0%) | 4,002/4,002 (100.0%) | 2,997/2,997 (100.0%) |
| Pulmonary circulation disorders | 6,999/6,999 (100.0%) | 4,002/4,002 (100.0%) | 2,997/2,997 (100.0%) |
| Renal failure | 6,999/6,999 (100.0%) | 4,002/4,002 (100.0%) | 2,997/2,997 (100.0%) |
| Solid tumor without metastasis | 6,999/6,999 (100.0%) | 4,002/4,002 (100.0%) | 2,997/2,997 (100.0%) |
| Valvular heart disease | 6,999/6,999 (100.0%) | 4,002/4,002 (100.0%) | 2,997/2,997 (100.0%) |
| Weight loss | 6,999/6,999 (100.0%) | 4,002/4,002 (100.0%) | 2,997/2,997 (100.0%) |
| Clinical comorbidities | | | |
| Acute kidney injury | 6,999/6,999 (100.0%) | 4,002/4,002 (100.0%) | 2,997/2,997 (100.0%) |
| Asthma | 6,999/6,999 (100.0%) | 4,002/4,002 (100.0%) | 2,997/2,997 (100.0%) |
| Atrial fibrillation | 6,999/6,999 (100.0%) | 4,002/4,002 (100.0%) | 2,997/2,997 (100.0%) |
| Chronic kidney disease | 6,999/6,999 (100.0%) | 4,002/4,002 (100.0%) | 2,997/2,997 (100.0%) |
| Chronic obstructive pulmonary disease | 6,999/6,999 (100.0%) | 4,002/4,002 (100.0%) | 2,997/2,997 (100.0%) |
| Deep vein thrombosis | 6,999/6,999 (100.0%) | 4,002/4,002 (100.0%) | 2,997/2,997 (100.0%) |
| Dementia | 6,999/6,999 (100.0%) | 4,002/4,002 (100.0%) | 2,997/2,997 (100.0%) |
| Diabetes mellitus | 6,999/6,999 (100.0%) | 4,002/4,002 (100.0%) | 2,997/2,997 (100.0%) |
| End-stage kidney disease | 6,999/6,999 (100.0%) | 4,002/4,002 (100.0%) | 2,997/2,997 (100.0%) |
| Epilepsy | 6,999/6,999 (100.0%) | 4,002/4,002 (100.0%) | 2,997/2,997 (100.0%) |
| Hypertension | 6,999/6,999 (100.0%) | 4,002/4,002 (100.0%) | 2,997/2,997 (100.0%) |
| Ischemic heart disease | 6,999/6,999 (100.0%) | 4,002/4,002 (100.0%) | 2,997/2,997 (100.0%) |
| Malignancy (any) | 6,999/6,999 (100.0%) | 4,002/4,002 (100.0%) | 2,997/2,997 (100.0%) |
| Myocardial infarction | 6,999/6,999 (100.0%) | 4,002/4,002 (100.0%) | 2,997/2,997 (100.0%) |
| Pulmonary embolism | 6,999/6,999 (100.0%) | 4,002/4,002 (100.0%) | 2,997/2,997 (100.0%) |
| Rheumatoid arthritis | 6,999/6,999 (100.0%) | 4,002/4,002 (100.0%) | 2,997/2,997 (100.0%) |
| Sickle cell disease | 6,999/6,999 (100.0%) | 4,002/4,002 (100.0%) | 2,997/2,997 (100.0%) |
| Stroke | 6,999/6,999 (100.0%) | 4,002/4,002 (100.0%) | 2,997/2,997 (100.0%) |
| Systemic lupus erythematosus | 6,999/6,999 (100.0%) | 4,002/4,002 (100.0%) | 2,997/2,997 (100.0%) |
| Antibiotics | | | |
| Amikacin | 6,103/6,999 (87.2%) | 3,807/4,002 (95.1%) | 2,296/2,997 (76.6%) |
| Amoxicillin-clavulanate | 2,412/6,999 (34.5%) | 2,412/4,002 (60.3%) | 0/2,997 (0.0%) |
| Ampicillin | 528/6,999 (7.5%) | 528/4,002 (13.2%) | 0/2,997 (0.0%) |
| Ampicillin-sulbactam | 2,152/6,999 (30.7%) | 1,523/4,002 (38.1%) | 629/2,997 (21.0%) |
| Aztreonam | 2,184/6,999 (31.2%) | 1,705/4,002 (42.6%) | 479/2,997 (16.0%) |
| Cefazolin | 461/6,999 (6.6%) | 461/4,002 (11.5%) | 0/2,997 (0.0%) |
| Cefepime | 5,860/6,999 (83.7%) | 3,262/4,002 (81.5%) | 2,598/2,997 (86.7%) |
| Cefotaxime | 1,918/6,999 (27.4%) | 1,493/4,002 (37.3%) | 425/2,997 (14.2%) |
| Cefoxitin | 1,804/6,999 (25.8%) | 1,804/4,002 (45.1%) | 0/2,997 (0.0%) |
| Ceftazidime | 5,940/6,999 (84.9%) | 3,250/4,002 (81.2%) | 2,690/2,997 (89.8%) |
| Ceftriaxone | 768/6,999 (11.0%) | 582/4,002 (14.5%) | 186/2,997 (6.2%) |
| Cefuroxime | 1,387/6,999 (19.8%) | 1,387/4,002 (34.7%) | 0/2,997 (0.0%) |
| Ciprofloxacin | 6,436/6,999 (92.0%) | 3,760/4,002 (94.0%) | 2,676/2,997 (89.3%) |
| Ertapenem | 2,210/6,999 (31.6%) | 2,210/4,002 (55.2%) | 0/2,997 (0.0%) |
| Gentamicin | 5,176/6,999 (74.0%) | 3,607/4,002 (90.1%) | 1,569/2,997 (52.4%) |
| Imipenem | 6,263/6,999 (89.5%) | 3,602/4,002 (90.0%) | 2,661/2,997 (88.8%) |
| Levofloxacin | 4,983/6,999 (71.2%) | 2,885/4,002 (72.1%) | 2,098/2,997 (70.0%) |
| Meropenem | 6,041/6,999 (86.3%) | 3,567/4,002 (89.1%) | 2,474/2,997 (82.5%) |
| Minocycline | 510/6,999 (7.3%) | 348/4,002 (8.7%) | 162/2,997 (5.4%) |
| Piperacillin-tazobactam | 5,118/6,999 (73.1%) | 3,499/4,002 (87.4%) | 1,619/2,997 (54.0%) |
| Tigecycline | 2,275/6,999 (32.5%) | 2,274/4,002 (56.8%) | 1/2,997 (0.0%) |
| Tobramycin | 4,056/6,999 (58.0%) | 2,222/4,002 (55.5%) | 1,834/2,997 (61.2%) |
| Trimethoprim-sulfamethoxazole | 5,369/6,999 (76.7%) | 3,734/4,002 (93.3%) | 1,635/2,997 (54.6%) |
| Abbreviations: GNB, gram-negative bacilli; HIV/AIDS, human immunodeficiency virus/acquired immunodeficiency syndrome; ICU, intensive care unit. | | | |
| a—Completeness shown as nonmissing values / denominator (%). | | | |
